# Wdr68 Mediates Dorsal and Ventral Patterning Events for Craniofacial Development

**DOI:** 10.1371/journal.pone.0166984

**Published:** 2016-11-23

**Authors:** Estibaliz Alvarado, Mina Yousefelahiyeh, Greg Alvarado, Robin Shang, Taryn Whitman, Andrew Martinez, Yang Yu, Annie Pham, Anish Bhandari, Bingyan Wang, Robert M. Nissen

**Affiliations:** Department of Biological Sciences, California State University Los Angeles, Los Angeles, California, United States of America; University of Maryland Center for Environmental Science, UNITED STATES

## Abstract

Birth defects are among the leading causes of infant mortality and contribute substantially to illness and long-term disability. Defects in Bone Morphogenetic Protein (BMP) signaling are associated with cleft lip/palate. Many craniofacial syndromes are caused by defects in signaling pathways that pattern the cranial neural crest cells (CNCCs) along the dorsal-ventral axis. For example, auriculocondylar syndrome is caused by impaired Endothelin-1 (Edn1) signaling, and Alagille syndrome is caused by defects in Jagged-Notch signaling. The BMP, Edn1, and Jag1b pathways intersect because BMP signaling is required for ventral *edn1* expression that, in turn, restricts *jag1b* to dorsal CNCC territory. In zebrafish, the scaffolding protein Wdr68 is required for *edn1* expression and subsequent formation of the ventral Meckel’s cartilage as well as the dorsal Palatoquadrate. Here we report that *wdr68* activity is required between the 17-somites and prim-5 stages, that *edn1* functions downstream of *wdr68*, and that *wdr68* activity restricts *jag1b*, *hey1*, and *grem2* expression from ventral CNCC territory. Expression of *dlx1a* and *dlx2a* was also severely reduced in anterior dorsal and ventral 1^st^ arch CNCC territory in *wdr68* mutants. We also found that the BMP agonist isoliquiritigenin (ISL) can partially rescue lower jaw formation and *edn1* expression in *wdr68* mutants. However, we found no significant defects in BMP reporter induction or pSmad1/5 accumulation in *wdr68* mutant cells or zebrafish. The Transforming Growth Factor Beta (TGF-β) signaling pathway is also known to be important for craniofacial development and can interfere with BMP signaling. Here we further report that TGF-β interference with BMP signaling was greater in *wdr68* mutant cells relative to control cells. To determine whether interference might also act in vivo, we treated *wdr68* mutant zebrafish embryos with the TGF-β signaling inhibitor SB431542 and found partial rescue of *edn1* expression and craniofacial development. While ISL treatment failed, SB431542 partially rescued *dlx2a* expression in *wdr68* mutants. Together these findings reveal an indirect role for Wdr68 in the BMP-Edn1-Jag1b signaling hierarchy and dorso-anterior expression of *dlx1a/2a*.

## Introduction

Birth defects are among the leading causes of infant mortality and contribute substantially to illness and long-term disability. Craniofacial anomalies, excluding cleft lip and palate, occur in 1 out of every 1600 births in the United States [1]. Defects in Bone Morphogenetic Protein (BMP) signaling are associated with cleft lip/palate [2, 3]. Many craniofacial syndromes are caused by defects in signaling pathways that pattern the cranial neural crest cells (CNCCs) along the dorsal-ventral (D/V) axis. For example, Auriculocondylar syndrome is caused by impaired Endothelin-1 (Edn1) signaling [4–6] and Alagille syndrome is caused by defects in Jagged (Jag)-Notch signaling [7, 8]. The BMP, Edn1, and Jag1b pathways intersect because BMP signaling is required for ventral *edn1* expression that, in turn, restricts *jag1b* to dorsal CNCC territory [9–11].

Craniofacial development in vertebrate organisms is a highly conserved process and involves interactions between multiple signaling pathways. The zebrafish *Danio rerio* is a model vertebrate organism particularly suited to the study of early developmental events. Craniofacial development begins shortly after the end of gastrulation with the specification and subsequent migration of CNCCs from the dorsal part of the neural tube. The CNCCs migrate ventro-laterally from the dorso-medial neural tube to one of seven pharyngeal arches. Once in the arches, the CNCCs receive and participate in multiple signaling events [12]. Lateral ectoderm and medial endoderm cells together form pouches that cup the CNCCs and deliver various paracrine signals important for both the survival and the patterning of the adjacent CNCCs [13–15]. The zebrafish jaw structures derived from the first arch CNCCs are the dorsal palatoquadrate (PQ) and ventral Meckel’s (M) cartilages [16, 17].

BMP and Jag-Notch signaling regulate ventral and dorsal CNCC patterning, respectively [9–11]. Edn1 signaling is required for ventral CNCC patterning downstream of BMP signaling [10, 18]. The combined action of these signaling pathways sub-define regions of CNCCs along the D/V axis within the first pharyngeal arch. These sub-defined territories are evident in the layered expression patterns of the distal-less (dlx) family of transcription factors [19]. Specifically, loss of Edn1 signaling results in the loss or reduction of ventral and intermediate structures such as Meckel’s cartilage (M) and the jaw joint. Additionally, the loss of Edn1 signaling has been shown to result in the dorsalization of ventral structures [18, 20–22]. In mice, mutants for either *edn1*^*-/-*^ or *ednra*^*-/-*^ display loss of expression for *dlx3-6*, and *hand2* [21, 22]. Similarly, *edn1*^*-/-*^ mutant zebrafish lose ventral and intermediate expression of *dlx3-6* and *hand2* [18]. Overexpression of Bmp4 expands ventral territory via upregulation of *edn1* and the corresponding downstream network of transcription factors including *hand2* and several *dlx* genes [10]. Conversely, disruption of later stage Bmp signaling by overexpressing a dominant negative Bmp receptor after CNCC induction results in the loss of ventral *edn1* and *dlx6a* expression, intermediate-ventral *dlx3b* expression, and the ventral expansion of the normally dorsal restricted *jag1b* [9]. In zebrafish, *jag2* and *jag1b* are expressed in the CNCCs and pharyngeal pouch endoderm [11, 23] and antisense knockdown of them results in reductions in dorsal cartilages [24]. Zebrafish *jag1b* mutants display dorso-posterior defects in PQ formation; antisense morpholino knockdowns of *notch2* yield a similar phenotype consistent with a traditional Jag-Notch signaling requirement for dorsal development. Loss of Jagged-Notch signaling results in the dorsal expansion of ventral and intermediate specifying *dlx3b* and *dlx5a* markers. Misexpression of *jag1b* results in the dorsalization of ventral structures, particularly with the transformation of M into a more PQ like structure. *Jag1b* is normally restricted to the dorsal-most CNCC within the first arch, however ubiquitous overexpression of *jag1b* results in the loss of ventrally expressed *hand2* and *edn1* as well as restriction of ventral-intermediate markers *dlx3b*, *dlx5a*, and *dlx6a* to the most ventral territory of the arch [11]. Taken together, these interactions yield a model in which BMP signaling patterns ventral and intermediate territory at least partly through induction of Edn1 signaling, while Jag-Notch signaling patterns dorsal territory, and mutual antagonism stabilizes patterning along the D/V axis [25]. These combined interactions yield spatial restrictions of a nested network of transcription factors into dorsal, intermediate, and ventral territories that prefigure the future lower jaw, joint, and upper jaw cartilages.

BMP2/4/7 ligands are members of the Transforming Growth Factor Beta (TGF-β) superfamily of growth factors. The TGF-β signaling pathway is also important for several patterning events during early embryonic development, including proper craniofacial development [26]. While BMPs act through the phospho-Smad1/5 (pSmad1/5) transcription factors in complex with the common shared Smad4 to regulate downstream target gene expression, the TGF-β1/2/3 ligands instead act through pSmad2/3 also in complex with Smad4 [27, 28]. Interestingly, TGF-β can interfere with BMP signaling via Smad3 displacement of Smad4 from pSmad1/5 complexes [29, 30].

Wdr68/Dcaf7 (hereafter referred to as Wdr68) is a highly conserved 342 amino acid length member of the WD40 repeat domain family of proteins that are generally known to function as scaffolding proteins [31–33]. Vertebrate Wdr68 interacts with several protein kinases including several members of the Dual-specificity tyrosine phosphorylation-regulated kinase (Dyrk) gene family [34–38]. Dyrk1a maps to the Down Syndrome (DS) critical region of human chromosome 21 and overexpression of Dyrk1a is a major contributor to the neurodevelopmental defects present in DS patients [39, 40]. Dyrk1a and Dyrk1b possess RNApII-CTD kinase activity important for the expression of several genes [41]. High-throughput protein interaction assays also suggest that Wdr68 interacts with the pSmad2/3 components of TGF-β signaling [42]. The subcellular localization of Wdr68 is likely regulated. When expressed alone, Wdr68 is found in both the cytoplasm and nucleus. However, co-expression with the RNApII-CTD kinase Dyrk1a induces nuclear translocation of Wdr68 [35, 37, 43]. Wdr68 is expressed widely in early development and regulates ventral cartilage formation in the zebrafish [36, 43]. Specifically, *wdr68* is required for expression of *edn1* and genes downstream of Edn1 such as *bapx1*, *hand2*, and several *dlx* genes [36]. *wdr68* is also required for the expression of the *spaw*, *lft1*, and *lft2* genes [34] that are downstream of BMP signaling in the zebrafish [44, 45].

Together, these previous findings suggest Wdr68 may modulate TGF-β/BMP signaling to downstream targets such as the Edn1-Jag1b network for D/V patterning. The *wdr*68^hi3812^ allele contains a retroviral insertion within the first exon after codon 44 and is therefore considered a null allele [36, 46]. Here we report that the temperature-dependent severity of the cartilage defects present in *wdr*68^hi3812^ homozygotes is a consequence of differential perdurance of maternally derived Wdr68. We then exploited the temperature-dependence, along with a *Tg(hsp70l*:*GFP-Wdr68)* zebrafish line, to define a temporal window between the 17-somites and prim-5 stages during which *wdr68* activity is required for craniofacial development. Notably, this window overlaps substantially with the known onset of *edn1* expression and activity during craniofacial development. We also report that *edn1* expression can partially rescue *dlx6a* expression in *wdr68* mutants, consistent with it functioning downstream of *wdr68*. However, *edn1* expression was unable to restore *hand2* expression in *wdr68* mutants. We also found ventral expansion of the expression of the normally dorsal-restricted *jag1b*, *hey1*, and *grem2* genes in *wdr68* mutants. We further report pharmacogenetic interactions between BMP signaling and *wdr68*. Specifically, the BMP antagonist dorsomorphin (DM) exacerbates the *wdr68* cartilage and *edn1* expression defects while the BMP agonist isoliquiritigenin (ISL) partially rescues the *wdr68* cartilage and *edn1* expression defects. Using CRISPR/Cas9 generated mouse C2C12 sublines lacking *wdr68*, we found no significant changes in pSmad1/5 levels, pYap1 levels, or BRE-Luciferase responsiveness to BMP ligands. We also found little to no defects in pSmad1/5 levels in zebrafish *wdr68* mutants. However, co-challenge with TGF-β and BMP ligands revealed significant enhancement of TGF-β interference with BRE-Luciferase responsiveness to BMP ligands in C2C12 sublines lacking *wdr68*. Suggesting conservation of this mechanism in vivo, we also report that treating *wdr68* mutants with the TGF-β inhibitor SB431542 also partially rescued the craniofacial and *edn1* expression defects. Further analysis of dorsal CNCC markers revealed that expression of *dlx1a* and *dlx2a* was severely reduced in anterior dorsal and ventral 1^st^ arch CNCC territory in *wdr68* mutants. While ISL treatment failed to restore *dlx2a* expression, SB431542 partially rescued *dlx2a* in *wdr68* mutants. Together, the findings reported here reveal an indirect role for Wdr68 in the BMP-Edn1-Jag1b signaling hierarchy and dorso-anterior expression of *dlx1a/2a*. These findings also suggest interactions between Wdr68 and TGF-β signaling that will require further investigation.

## Materials and Methods

### Chemicals and reagents

Dorsomorphin (P5499, Sigma-Aldrich), isoliquiritigenin (I3766, Sigma-Aldrich), SB431542 (S4317, Sigma-Aldrich), BMP4 (HZ-1045, Humanzyme), and TGF-β1 (HZ-1011, Humanzyme) were purchased and used according to the manufacturers recommendations. Antibodies used were anti-Wdr68 (HPA022948, Sigma-Aldrich), anti-β-tubulin (sc-55529, Santa Cruz Biotechnology Inc.), anti-pYap1 (13008S, Cell Signaling), anti-Yap1 (4912S, Cell Signaling), anti-pSmad1/5 (9511S, Cell Signaling), goat anti-mouse IgG-HRP (sc-2005, Santa Cruz Biotechnology Inc.), Amersham ECL anti-rabbit IgG, HRP-linked whole antibody (from donkey) (NA934, GE Healthcare).

### Zebrafish husbandry

This project was approved and conducted under the approved Cal State LA IACUC protocol (14–2 Renewal 11–3) and the zebrafish animals (TAB14 and AB* backgrounds) used in it were reared in strict accordance with IACUC guidelines. Zebrafish embryos were raised at 24°C, 28.5°C, or 32°C as specified in specific experiments.

### Alcian blue staining

Alcian blue staining was performed as previously described [34, 43]. Briefly, after fixation with 4% PFA, embryos were washed in Phosphate Buffered Saline + 0.1% Tween-20 (PBST) and dehydrated through 50% PBST/ 50% Methanol (MeOH) while rocked for 5 minutes. Samples were put into 100% Methanol and stored on ice for 30 minutes. Methanol was then removed and samples stained in 0.1% Alcian Blue overnight while rocking. The next day samples were rinsed twice with 100% Ethanol (EtOH) and then rehydrated through 50% PBST/ 50% MeOH into PBST. Samples were digested in 0.05% Trypsin in saturated Sodium Borate (Na_2_B_4_O_7_) for 3 hours at 37°C. Samples were bleached overnight in a solution of 3% H_2_O_2_; 1% KOH at 4°C. The next day samples were washed in PBS + 1.0% Tween-20 for 5 min and then re-suspended in 80% glycerol; 0.1% Tween-20. Animals were scored for the presence of the M and PQ cartilages.

### Immunofluorescence

Embryos were fixed in 4% PFA in PBST for 2 hours at room temperature (RT), then washed twice with PBST, once with 50% PBST/50% MeOH, once with ice-cold 100% MeOH for 2 min, quickly washed twice with dH_2_O and then exposed to 100% acetone at -20°C for 4 min followed by three washes with PBST. Embryos were then washed twice with PBSTTD (PBST + 0.1% Triton X-100 + 1% DMSO) for 5 min. Embryos and primary antibodies were pre-blocked for 30 min at RT in Block solution (PBSTTD + 0.5% Boehringer Mannheim Block solution + 10% Lamb serum) and then incubated in primary antibody overnight at 4°C. The next day embryos were washed twice quickly and five times for 10 minutes each in PBSTD (PBST + 1% DMSO) and then re-blocked for 30 minutes before exposure to blocked secondary antibody for 2 hours at RT. Then, embryos were washed twice quickly and five times for 10 minutes each in PBSTD followed by imaging on a Zeiss Apotome microscope.

### Confocal imaging

The *Tg(sox10*:*mCherryCAAX)* animals were kindly provided by the Crump Lab [47]. Confocal multi-TIFF stacks were captured using an Olympus IX81 confocal microscope on agarose-mounted embryos and analyzed using FIJI volume viewer.

### Transgenesis constructs and isolation of transgenic lines

The *Tg(hsp70l*:*GFP-wdr68*)^csu6^ and *Tg(hsp70l*:*GFP-wdr68*)^csu9^ lines were created using the Gateway Tol2kit reagents [48]. Briefly, the *GFP-wdr68* fusion fragment from pCS2+GFP*-wdr68* [36] was inserted into the pDONR221 plasmid to yield the pME-GFP*-wdr68* plasmid that was then combined with p5E-hsp70l, p3E-polyA and pDestTol2CG2 to create pT2-hsp70l:*GFP-wdr68*-CG2. This plasmid was then co-injected with Tol2 transposase mRNA into zebrafish embryos at the 1-cell stage to create the founders for subsequent isolation of stable transgenic lines.

### Microinjections

Zebrafish embryos were harvested and injected with morpholino solution at the one to four cell stages as previously described [34, 43]. The morpholino solutions used were 3812-2/4: 200μM 3812–4 morpholino, 500μM 3812–2 morpholino in 0.1% phenyl red; 0.5x PBS or 3812–1: 700μM 3812–1 morpholino in 0.1% phenyl red; 0.5x PBS [36]. The 3812-2/4 solution blocks endogenous wdr68 mRNA translation, while the 3812–1, which does not block translation, served as a negative control.

### Heat Shock Rescues

*Tg(hsp70l*:*GFP-wdr68)*^*csu9*^ animals were crossed to wildtype TAB14 fish. Embryos were collected at the one cell stage and injected with either 3812-2/4 morpholino, blocking translation of endogenous Wdr68, or with the negative control 3812–1 morpholino. Embryos were then allowed to develop in a 28.5°C incubator. Heat shocks were performed at 39°C for 30min at each of the stages of interest. Heat shocks were conducted at 5-somites, 12-somites, 15-somites, 17-somites, 20-somites, 25-somites, prim-5, prim-12 and prim-20 stages. After 30 minutes of heat shock, animals were returned to 28.5°C, and allowed to develop until the formation of the swim bladder was observed in control animals [approximately 5 days post fertilization (dpf)]. Animals were then fixed for 1 hour at room temperature or overnight at 4°C in 4% PFA in PBST, and cartilages scored via Alcian Blue Staining.

### Probe Synthesis and In Situ Hybridization

Probes for *jag1b*, *hey1*, and *grem2* were previously described [10, 11]. The *edn1* plasmid has been previously described [18]. The *dlx6a*, *hand2*, *dlx1a*, and *dlx2a* plasmids have been previously described [49–52]. All transcription reactions were conducted with the MEGAscript SP6, T7, or T3 Transcription Kits per manufacturer’s instructions (Ambion), with the adaptation of using digoxigenin-labeled-UTP. ISH was performed as previously described [34, 53] with the following modifications. The *jag1b*, *hey1*, *grem2*, and *dlx1a* probes were used at 0.2ng/μL in hybridization buffer (hyb) overnight at 65°C. The *edn1*, *hand2*, *dlx6a*, and *dlx2a* probes were used at 0.5ng/μL in hyb buffer overnight at 70°C. Once development was complete, embryos were washed in PBST, bleached in 10% H_2_O_2_; 5% formamide in PBST for 20 min. Finally embryos were washed in PBST and cleared in 80% glycerol containing 0.1% Tween-20 in dH_2_O for imaging.

### Embryo Genotyping

Embryos were separated into individual Eppendorf tubes, excess water removed, and then digested in Proteinase K at a final concentration of 100μg/ml for 2 hours at 55°C and were vortexed every 15 minutes during the incubation. Samples were then diluted two-fold with dH_2_O and vortexed for 1 minute. Proteinase K was then inactivated by heating samples to 96°C for 15 minutes. Samples were then centrifuged at top speed for 1 minute to pellet cellular debris. 2μL of the solution was then used as template in a PCR reaction using the previously described primers 3812-c; 3812–3; and LTR-f1 [36].

### Plasmid construction and plasmid rescue assay

The primers edn1-f1 5’-TTCTTCGGATCCACCATGCATTTGAGGATTATTTTCCCAGTTCTG-3’ and edn1-r1 5’-TTCTTCGAATTCCTATGAGTTTTCAGAAATCCACGCTTG-3’ were used to PCR amplify the *edn1CDS* from a previously described ISH probe plasmid containing *edn1* [18]. The insert was TOPO cloned, DNA sequence verified, and subcloned into pCS2+ to yield pCS2+edn1CDS. For rescue, a pCS2+eGFP marker plasmid was co-injected at the 1-cell stage with either pCS2+dsRed or the pCS2+edn1CDS plasmid, each at 50ng/μL.

### Drug Treatments of Embryos

Adult *wdr68*^*hi3812/+*^ zebrafish were crossed to obtain groups of embryos that were placed in a 24°C incubator for later dorsomorphin (DM) treatment or a 32°C incubator for later isoliquiritigenin (ISL) or SB431542 (SB) treatment. Dechorionated embryos were exposed to 0.1% DMSO control or DM/ISL/SB starting at the 14- to 15-somites stage and processed at 5 dpf for Alcian staining or at the 20–24 somites stages for ISH analysis. Various drugs were kept on the embryos until fixation.

### CRISPR/Cas9-mediated knockouts in C2C12 cells

C2C12 cells were obtained from the ATCC. pLentiCRISPRv2-dcaf7-2 was generated by digesting the pLentiCRISPRv2 plasmid with BsmBI, the oligonucleotides CRISPR-mdcaf7-2f: 5’-CACCGACATCGCCTTCAGCCGCGC-3’ and CRISPR-mdcaf7-2r: 5’-AAACGCGCGGCTGAAGGCGATGTC-3’ were annealed, and then ligated into the vector fragment as previously described [54]. Putative clones were isolated from Stbl3 competent cells and verified by DNA sequencing. Lentiviral particles were generated by co-transfecting 293T cells with the virus packaging plasmids psPAX2 and pCMV-VSV-G along with pLentiCRISPR-dcaf7-2. Cleared virus-containing supernatant was used to transduce C2C12 cells in C2C12 growth medium (GM) (DMEM + 2.5% FBS + 10% calf serum + 7.4 mM L-glutamine + 100μg/mL pen/strep). The transduced C2C12 cells were subjected to 1μg/mL puromycin (GM+puro) selection, and clonal sublines were isolated by serial dilution. Putative knock-out sublines were screened by Western blot to identify functional knock-outs.

### Western blots on zebrafish embryo or C2C12 cell extracts

Embryos were ground on ice in a 1.5 mL microcentrifuge tube using a plastic pestle in 50μL ice-cold RIPA buffer (50mM Tris pH 8, 150mM NaCl, 1% Igepal CA630, 0.5% NaDeoxycholate, 0.1% SDS) with 1x Protease Inhibitor Cocktail (PIC). The samples were vortexed for 1 minute and then centrifuged for 1 minute at room temperature at high speed. The samples were maintained on ice for 1 hour and every 10 minutes were vortexed for 1 minute. After the 1-hour period ended, the tubes were centrifuged to pellet debris and supernatants quantified by Bradford assay prior to being subjected to western blot analysis as described further below. C2C12 cell extracts were made from 10cm plates of cells grown in GM or Differentiation Medium (DMEM + 2% Horse serum + 100ug/mL pen/strep), rinsed twice with ice-cold PBS, and then incubated in 0.5mL ice-cold RIPA buffer + 1x PIC for 15 min at 4°C. Cells were then scraped from the plate, incubated on ice for 1 hour, centrifuged at 10,000g at 4°C and supernatants quantified by Bradford assay prior to being subjected to western blot analysis as follows. Equal amounts of protein samples were boiled for 5 minutes at 95°C, ran on 8–16% SDS-PAGE gels, and then transferred onto PVDF membrane (Thermo Scientific). The PVDF membrane was blocked overnight at 4°C with 5% non-fat dry milk in PBST (PBS+0.01% Tween-20) with 0.02% NaN_3_. The following day the blocking buffer was removed and blocked primary antibody was added. The membrane was then rinsed 3X for 5 minutes with PBST and then blocked secondary antibody was added. After the incubation period the membrane was rinsed 3X for 5 minutes with PBST. In most cases, the same blot was stripped and re-probed for relevant controls (beta-tubulin, total Yap).

### Transient transfections and reporter assays

BMP-luciferase (BRE-Luc) reporter plasmid [55] and SV-40 Renilla Luciferase (SV40-Luc) reporter plasmid were co-transfected into the C2C12 sublines using X-tremeGene HP as per the manufacturers recommendations (Roche). After 8 or 16 hours of transfection, DNA-lipid complexes were replaced with fresh medium -/+ BMP4 and/or -/+ TGF-β1. After an additional 8 or 20 hours, cell extracts were harvested for luminometer measurements using the Dual-luciferase reporter (DLR) assay kit reagents (Promega).

### Statistical Analysis

Pairwise comparisons were performed using the Student’s T-test. Experiments containing 3 or more conditions were subjected to one-way ANOVA and post-hoc Tukey HSD tests.

## Results

### *wdr68* activity is required during a window between the 17 somites and prim-5 stages

**Fig 1:**
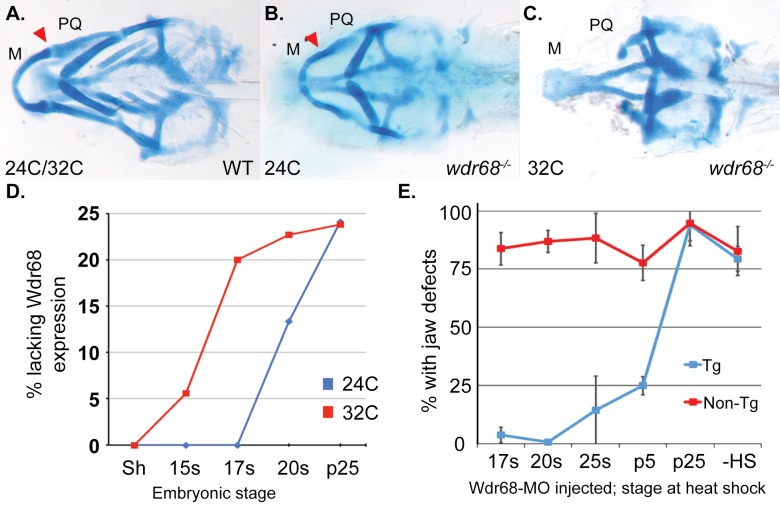
Wdr68 is required for craniofacial development between the 17 somites and prim-5 stages. A) Ventral view of 5dpf alcian blue stained cartilages in wild type zebrafish. M: Meckel’s PQ: Palatoquadrate. B) Mild mutant phenotype resulting from rearing of embryos at 24°C, characterized by joint fusions (arrowhead) between M and PQ. C) Severe mutant phenotype resulting from rearing of embryos at 32°C, characterized by the loss of M and PQ. D) Immunohistochemistry readily detects maternal Wdr68 protein up to the 17 somites stage in *wdr68*^*hi3812/hi3812*^ mutants raised at the permissive 24°C temperature (blue line). Maternal Wdr68 is lost by the 17 somites stage in *wdr68*^*hi3812/hi3812*^ mutants raised at the non-permissive 32°C temperature (red line). E) *wdr68-MO* injected non-transgenic (Non-Tg) animals display jaw defects regardless of heat shock. *wdr68-MO* injected *Tg(hsp70l*:*GFP-Wdr68)*^*csu9*^ animals that are heat shocked by the prim-5 stage show rescue from jaw defects. Error bars indicate standard deviation. Additional abbreviations: Shield (Sh), 15 somites (15s), 17 somites (17s), 20 somites (20s), 25 somites (25s), prim-5 (p5), prim-25 (p25), no heat shock (-HS).

The *wdr68*^*hi3812/hi3812*^ mutant was originally described as presenting a range of jaw defects that varied from only mild joint fusions to near complete losses of the palatoquadrate (PQ) and Meckel’s (M) cartilages [36]. The initial characterization was completed using embryos raised at the standard 28.5°C. We have since found that rearing the animals at 24°C yielded mutant animals that mostly present only the mild M-PQ joint fusion defects (Fig 1B red arrowhead, compare to 1A). Specifically at 24°C, 79% of mutants presented only mild M-PQ fusions versus 21% displaying severe loss of the M and/or PQ cartilage. In contrast, rearing embryos at 32°C yielded mutant animals that mostly present severe losses of the M and PQ cartilages (Fig 1C compare to 1A, 1B). Specifically at 32°C, 6% of mutants presented only mild M-PQ fusions versus 94% displaying severe loss of the M and/or PQ cartilage. Because the *wdr68*^*hi3812*^ allele is a retroviral insertion within exon-1 near a splicing junction [36], and insertional mutations can cause temperature-dependent splicing defects [56], we examined animals reared at 24°C versus 32°C by RT-PCR in an effort to detect potential alternative splicing products but found no evidence to support such a mechanism (data not shown).

We next used immunofluorescence to determine whether Wdr68 protein might exhibit temperature-dependent differences in perdurance (Fig 1D and S1A–S1D Fig). We expected to find Wdr68 protein in very early stage *wdr68*^*hi3812/hi3812*^ mutants because we previously reported that *wdr68* transcripts are maternally supplied [36]. Consistent with that expectation, 100% of shield-stage embryos obtained from *wdr68*^*hi3812/+*^ in-crosses that were analyzed by immunofluorescence were positive for Wdr68 expression when raised at either 24°C or 32°C. Random genotyping identified 2/16 *wdr68*^*hi3812/hi3812*^ mutants confirming that the appropriate breeding cross had been made. However, by the 17-somites stage embryos raised at 32°C were strikingly different than those raised at 24°C (Fig 1D, 17s red versus blue). While no embryos raised at 24°C displayed severe loss of Wdr68 expression, a near-mendelian ratio (20%) of embryos raised at 32°C lacked Wdr68 expression. Genotyping confirmed that 5/5 embryos raised at 32°C and lacking Wdr68 expression by the 17-somites stage were *wdr68*^*hi3812/hi3812*^ mutants. Likewise, 9/9 embryos raised at 32°C and possessing Wdr68 expression were wildtype or *wdr68*^*hi3812/+*^ (hereafter summarized as +/*). Random genotyping of the embryos raised at 24°C that all had Wdr68 expression identified 3/10 *wdr68*^*hi3812/hi3812*^ mutants confirming that the appropriate breeding cross had been made. By the prim-25 stage, both embryos raised at 24°C and 32°C yielded near-mendelian ratios (24.1% and 23.8%, respectively) of embryos lacking Wdr68 expression. Thus, a critical function for M and PQ formation is performed during late somitogenesis by Wdr68 that is cut short in embryos raised at 32°C.

To further delineate the temporal window during which Wdr68 functions to mediate M and PQ formation, we generated *Tg(hsp70l*:*GFP-wdr68)* zebrafish lines (S1E–S1G Fig). The lines were first characterized by heat shocking them at 39°C for various periods of time followed by fluorescence imaging (S1E and S1F Fig) and then harvested for western blot analysis using anti-Wdr68 antibody (S1G Fig). We found that a 30-minute heat shock was sufficient to yield fusion protein expression that lasted for at least 7 more hours (S1F and S1G Fig, lane 7). We then injected embryos from outcrosses of the *Tg*(*hsp70l*:*GFP-wdr68*) adults with a previously described antisense *wdr68-MO* [36] and subjected them to 30-minute heat shocks at various developmental stages to determine how late in development restored Wdr68 activity could still rescue craniofacial development (Fig 1E). Overexpression of GFP-Wdr68 in otherwise wildtype embryos did not yield any observable defects (S2A and S2B Fig). Over 75% of non-transgenic *wdr68-MO* injected sibling embryos displayed jaw defects in spite of receiving heat-shocks at all developmental time-points examined indicating that heat shock alone was unable of rescue (Fig 1E, red line non-Tg). These embryos were also indistinguishable from both transgenic and non-transgenic *wdr68-MO* injected embryos that did not receive any heat shock (Fig 1E, -HS data point at the far right for each line; S2C and S2D Fig representative images for mild and severe phenotypes). In contrast, less than 25% of *Tg(hsp70l*:*GFP-wdr68)* sibling embryos heat shocked at 39°C between the 17-somites and prim-5 stages displayed jaw defects indicating rescue of craniofacial development as late as the prim-5 stage (Fig 1E, blue line; S2G Fig compare 25s and p5 with p25 and -HS). However, heat shocking the *wdr68-MO-*injected *Tg(hsp70l*:*GFP-wdr68)* embryos at the prim-25 stage failed to rescue craniofacial development (Fig 1E, blue line; S2G Fig). Thus, restoring Wdr68 function as late as the prim-5 stage is sufficient to rescue craniofacial development. Together these findings indicate that Wdr68 function is important for craniofacial development during a window between the 17-somites and prim-5 stages.

### *edn1* functions downstream of *wdr68*

**Fig 2:**
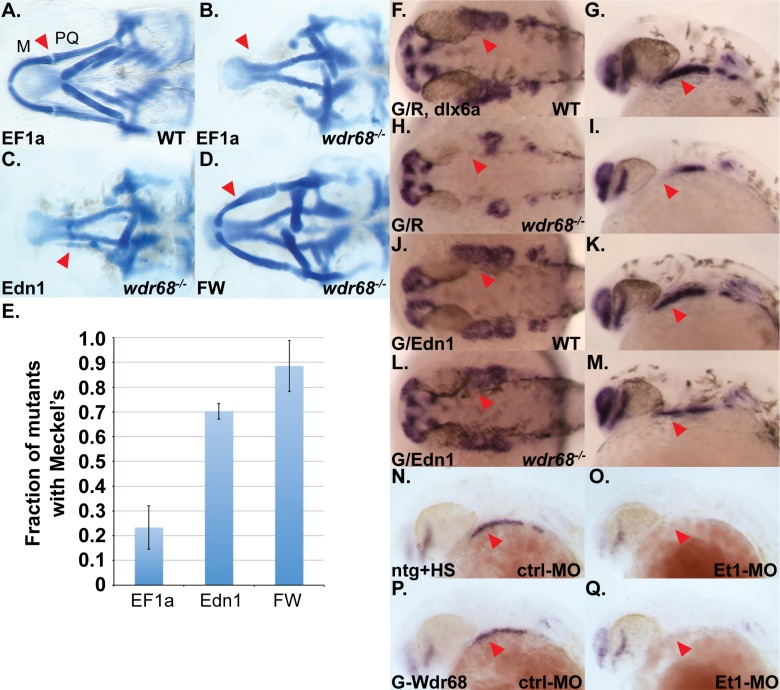
Edn1 functions downstream of *wdr68* for craniofacial development. (A-D) Ventral views of 5dpf Alcian stained craniofacial cartilages of zebrafish raised at 32°C. A) wildtype sibling injected with EF1a mRNA. B) *wdr68* mutant injected with EF1a mRNA. C) *wdr68*^*hi3812/hi3812*^ mutant injected with Edn1 mRNA. D) *wdr68*^*hi3812/hi3812*^ mutant injected with Flag-Wdr68 (FW) mRNA. E) Edn1 mRNA-injected mutants have more M cartilage elements than EF1a controls (p<0.001). (F-Q) ISH analysis of *dlx6a* expression in embryos raised at 32°C with red arrowhead pointing at 1^st^ arch CNCC territory. F, H, J, L) dorsal view of prim-5 stage. G, I, K, M) lateral view of prim-5 stage. F, G) wildtype sibling injected with GFP/dsRed (G/R) plasmid mix showing normal *dlx6a* expression. H, I) *wdr68*^*hi3812/hi3812*^ mutant injected with G/R plasmid mix showing loss of *dlx6a* in 1^st^ arch CNCC. J, K) wildtype sibling injected with GFP/Edn1 (G/Edn1) plasmid mix showing near-normal *dlx6a* expression. L, M) *wdr68*^*hi3812/hi3812*^ mutant injected with G/Edn1 plasmid mix showing partial rescue of *dlx6a* in 1^st^ arch CNCC. N-Q) lateral view of *dlx6a* expression in prim-5 stage embryos raised at 28.5°C. N) heat shocked wildtype sibling control with normal *dlx6a*. O) heat shocked wildtype sibling injected with Et1-MO showing loss of *dlx6a*. P) heat shock induced Tg(*hsp70l*:*GFP-Wdr68)* sibling control with normal *dlx6a*. Q) heat shock induced Tg(*hsp70l*:*GFP-Wdr68)* sibling injected with Et1-MO showing loss of *dlx6a*.

We previously showed that *edn1* expression depends on *wdr68* activity and that expression of downstream targets of Edn1 signaling, such as *dlx6a* and *hand2*, are also reduced or absent in *wdr68*^*hi3812/hi3812*^ mutants [36]. The simplest model for these observations is that *edn1* functions downstream of *wdr68* and that the primary function of *wdr68* in lower jaw development is, either directly or indirectly, the induction of *edn1* expression. A simple prediction of this model is that restoring *edn1* expression in a *wdr68*^*hi3812/hi3812*^ mutant should also restore the expression of *edn1* target genes, such as *dlx6a* and *hand2*. To test this model, we generated a plasmid construct for expressing *edn1*, injected embryos from crosses of *wdr68*^*hi3812/+*^ adults with various combinations of mRNAs or plasmids, and then processed the embryos raised at 32°C for either alcian blue staining of cartilages (Fig 2A–2E) or in situ hybridization (ISH) using *dlx6a* probe to assess potential rescue (Fig 2F–2Q). The negative control mRNA EF1a had no effect on wildtype siblings and failed to rescue *wdr68*^*hi3812/hi3812*^ mutants (Fig 2A and 2B). In contrast a significantly higher fraction of the embryos injected with Edn1 mRNA displayed partially restored M-like cartilages in *wdr68*^*hi3812/hi3812*^ mutants (Fig 2C and 2E; compare Edn1 vs EF1a, p<0.001). However, Edn1-injected *wdr68*^*hi3812/hi3812*^ mutants largely failed to form normal PQ-like cartilages. As expected, *wdr68*^*hi3812/hi3812*^ mutants injected with Flag-Wdr68 mRNA displayed nearly-normal M and PQ cartilages (Fig 2D and 2E; compare FW vs EF1a, p<0.001).

As control for ISH analysis, wildtype sibling embryos injected with plasmids expressing GFP and dsRed (G/R) displayed normal expression levels of *dlx6a* in the pharyngeal arches (Fig 2F and 2G, red arrowhead). Likewise, *wdr68*^*hi3812/hi3812*^ mutants injected with the G/R plasmid mix displayed the expected loss of 1^st^ arch *dlx6a* expression (Fig 2H and 2I, red arrowhead). Wildtype sibling embryos injected with plasmids expressing GFP and Edn1 *(*G/Edn1*)* were slightly smaller than normal but displayed near-normal expression levels of *dlx6a* in the pharyngeal arches (Fig 2J and 2K, red arrowhead). In contrast, *wdr68*^*hi3812/hi3812*^ mutants injected with the G/Edn1 plasmid mix partially restored *dlx6a* expression (compare Fig 2L and 2M with 2H and 2I). Genotyping confirmed that 0/12 mutants injected with the G/R plasmid mix had restored *dlx6a* expression (Fig 2H and 2I, 0% rescue), while 9/14 mutants injected with the G/Edn1 plasmid mix had restored *dlx6a* expression (Fig 2L and 2M, 64% rescue). Notably, the restoration of *dlx6a* expression appeared largely restricted to the ventral-most region of the 1^st^ arch in G/Edn1-injected *wdr68*^*hi3812/hi3812*^ mutants (compare Fig 2M to 2K). A significant caveat to these findings is that, at least in our hands, plasmid-based Edn1 is very broadly expressed in these experiments and thus the partial rescue may be the result of a non-local ectopic Edn1 source (S3E and S3F Fig).

To further explore the epistatic relationships between *edn1* and *wdr68*, we examined *dlx6a* expression in Tg(*hsp70l*:*GFP-Wdr68)* embryos injected with antisense morpholino knockdown of *edn1 (Et1-MO)* (Fig 2N–2Q). Wildtype sibling control embryos displayed normal *dlx6a* expression (Fig 2N, red arrowhead). As expected, wildtype sibling embryos injected with Et1-MO displayed severe loss of *dlx6a* expression in all pharyngeal arches (Fig 2O, red arrowhead). Heat shock induction of GFP-Wdr68 overexpression did not alter *dlx6a* expression in control embryos (Fig 2P) and failed to rescue *dlx6a* expression in Et1-MO animals (Fig 2Q). We also found that GFP-Wdr68 overexpression did not affect *edn1* expression (S3A and S3B Fig). Expression of *dlx6a* in Et1-MO;*wdr68*^*hi3812/hi3812*^ mutants was severely reduced in all pharyngeal arches similar to that observed in embryos only injected with Et1-MO (S3C and S3D Fig; Fig 2O). In contrast, alcian blue stained cartilages readily detected a PQ-like cartilage in Et1-MO animals that was absent in Et1-MO;*wdr68*^*hi3812/hi3812*^ mutants (S3G and S3H Fig). Thus, *edn1* appears to function downstream of *wdr68* to facilitate ventral cartilage development and *dlx6a* expression.

To further explore the regulatory relationships between *wdr68 and edn1*, we also examined *hand2* expression (S5I–S5P Fig). Expression of *hand2* is both *edn1-*dependent and BMP-dependent [9]. Wildtype sibling embryos injected with the G/R plasmid mix displayed normal expression levels of *hand2* (S3I and S3J Fig, red arrowhead). Likewise, *wdr68*^*hi3812/hi3812*^ mutants injected with the G/R plasmid mix displayed the expected loss of 1^st^ arch *hand2* expression (S3K and S3L Fig, red arrowhead). Wildtype sibling embryos injected with the G/Edn1 plasmid mix displayed near-normal expression levels of *hand2* (S3M and S3N Fig, red arrowhead). In contrast to that observed for *dlx6a*, *wdr68*^*hi3812/hi3812*^ mutants injected with the G/Edn1 plasmid mix failed to rescue *hand2* expression (compare S3O and S3P Fig with S3K and S3L Fig). Genotyping confirmed that 0/6 mutants injected with the G/Edn1 plasmid mix had restored *hand2* expression (S3O and S3P Fig, 0% rescue). Thus, while *edn1* is downstream of *wdr68*, more complex regulatory interactions exist for at least some *edn1* target genes, such as *hand2*, that require other *wdr68-*dependent functions for expression.

### *wdr68* restricts jagged-notch signaling to first arch dorsal territory

**Fig 3:**
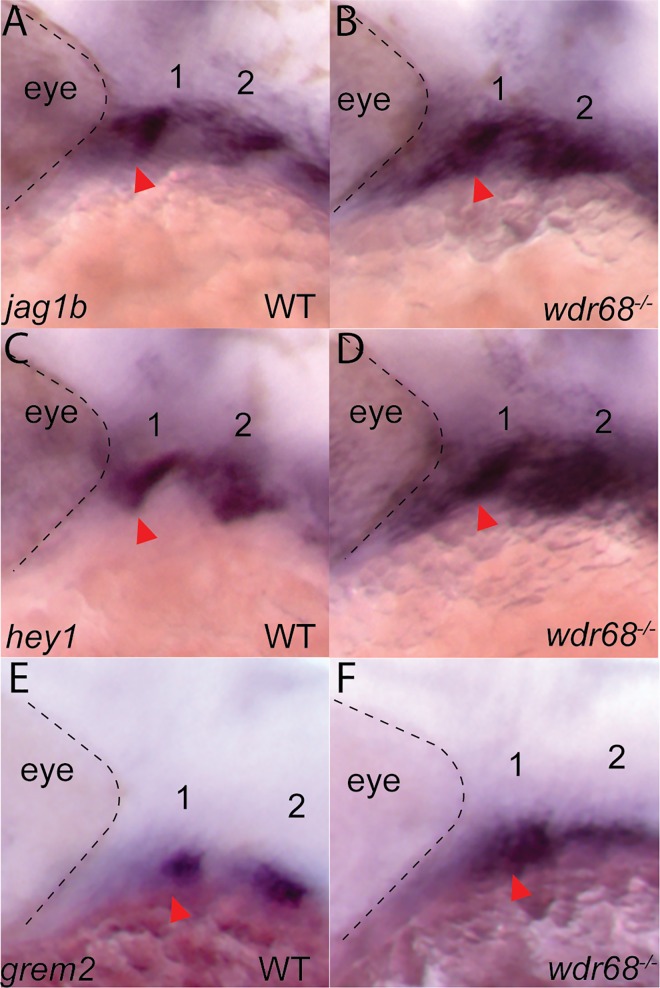
Wdr68 restricts expression of *jag1b*, *hey1*, and *grem2* from ventral territory. ISH analysis on embryos raised at 32°C. A) normal expression of *jag1b* in dorsal territory at the prim-25 stage, B) expansion of *jag1b* into ventral territory in *wdr68*^*hi3812/hi3812*^ mutants, C) normal expression of *hey1* in dorsal territory at the prim-25 stage, D) expansion of *hey1* into ventral territory in *wdr68*^*hi3812/hi3812*^ mutants, E) normal expression of *grem2* in dorsal territory at the prim-25 stage, F) expansion of *grem2* into ventral territory in *wdr68*^*hi3812/hi3812*^ mutants.

Signaling interactions between dorsally-restricted *jag1b* and *notch2* are important for the induction of similarly dorsally-restricted *hey1* and *grem2* expression [11]. Because *edn1* expression is known to be important for proper restriction of *jag1b*, *hey1*, and *grem2* expression from ventral territory, we examined their expression in embryos raised at 32°C from crosses of *wdr68*^*hi3812/+*^ adults. ISH revealed expansion of *jag1b* into the ventral territory of the first arch structure in *wdr68*^*hi3812/hi3812*^ mutants (compare Fig 3A to arrowhead in Fig 3B). Genotyping confirmed that 5/5 animals displaying the phenotype shown in Fig 3A were wildtype (+/*), while 6/6 animals displaying the phenotype depicted in Fig 3B were *wdr68*^*hi3812/hi3812*^ mutant animals. Expression of the transcription factor *hey1* depends on Jag-Notch signaling [11]. We also found expansion of *hey1* expression into the ventral territory of the first arch structure (compare Fig 3C to arrowhead in Fig 3D). Genotyping confirmed that 5/5 animals displaying the phenotype shown in Fig 3C were wildtype (+/*), while 5/5 animals displaying the phenotype depicted in Fig 3D were *wdr68*^*hi3812/hi3812*^ mutant animals. Expression of the normally dorsally-restricted BMP signaling antagonist *grem2* is also dependent on Jag-Notch signaling [10]. Mirroring our findings with *jag1b* and *hey1*, we found expansion of *grem2* expression into the ventral territory of the first arch structure (compare Fig 3E to arrowhead in Fig 3F). Genotyping confirmed that 5/5 animals displaying the phenotype shown in Fig 3E were wildtype (+/*), while 4/4 animals displaying the phenotype depicted in Fig 3F were *wdr68*^*hi3812/hi3812*^ mutant animals. Thus, we found that *wdr68* is required to restrict Jagged-Notch signaling events from ventral territory.

### A BMP antagonist exacerbates the severity of craniofacial defects and loss of *edn1* expression in *wdr68* mutants

**Fig 4:**
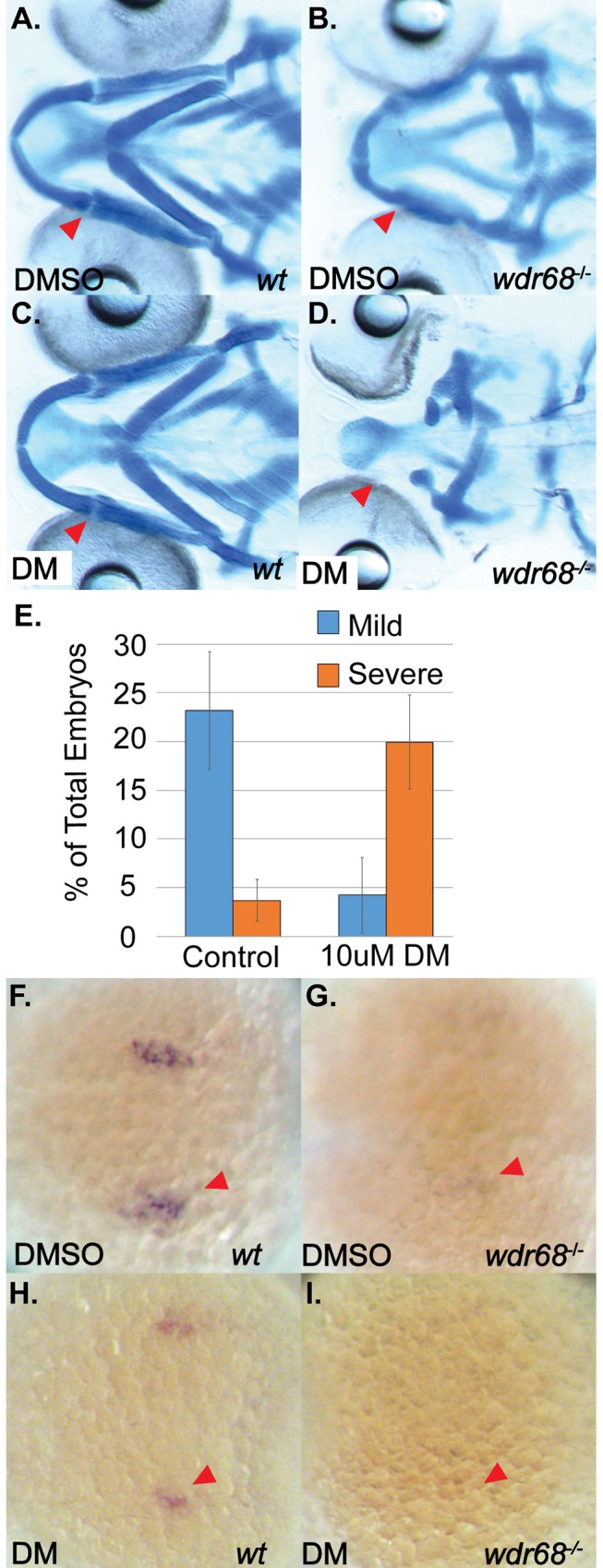
DM treatment induces loss of M cartilage and *edn1* expression in *wdr68*^hi3812/hi3812^ zebrafish. (A-D) Ventral views of 5dpf Alcian stained craniofacial cartilages of zebrafish raised at 24°C and treated with DMSO or 10μM DM at 14–15 somites stage. A) Wildtype embryo treated with DMSO control. B) *wdr68*^*hi3812/hi3812*^ mutant treated with DMSO show joint fusions between M and PQ. C) Wildtype embryo treated with DM reveal no defects in craniofacial cartilages. D) *wdr68*^*hi3812/hi3812*^ mutants treated with DM show severe reduction in PQ and deletion of M. E) DM-treated mutants show significantly more severe defects compared to the control group (p<0.040). (F-I) Dorsal views of *edn1* ISH analysis on 20 somites stage embryos treated with DMSO or 10μM DM starting at the 14–15 somites stage. F) Wildtype embryos treated with DMSO control. G) *wdr68*^*hi3812/hi3812*^ mutants treated with DMSO control show reduced *edn1* expression. H) Wildtype embryos treated with DM show mildly reduced *edn1* expression. I) *wdr68*^*hi3812/hi3812*^ mutants treated with DM lack *edn1* expression.

BMP signaling is required for *edn1* expression and ectopic BMP signaling can induce ectopic *edn1* expression [9, 10]. Thus, *edn1* is a downstream target of BMP signaling. We previously showed that *edn1* expression is also downstream of *wdr68* function [36] (and Fig 2). ISH analysis failed to detect any defects in BMP4 expression in *wdr68* mutants (S4 Fig), suggesting *wdr68* functions downstream or in parallel to BMP signaling. A simple model combining these observations is that *wdr68* facilitates BMP induction of *edn1*. If true, then treating *wdr68*^*hi3812/hi3812*^ mutants with a BMP antagonist will exacerbate loss of M formation and *edn1* expression. To test this, we treated embryos, from matings of *wdr68*^*hi3812/+*^ adult zebrafish, raised at 24°C with 10μM Dorsomorphin (DM) starting at the 14–15 somites stage. We chose to start treatments at this relatively late stage in order to avoid perturbing the very early developmental roles of BMP signaling. When grown at 24°C, *wdr68*^*hi3812/hi3812*^ embryos displayed only mild M-PQ joint fusions (see Fig 1B). We found that DMSO-treated wildtype (+/*) embryos exhibited normal cartilage formation while *wdr68*^*hi3812/hi3812*^ embryos exhibited M-PQ joint fusions (compare Fig 4A to 4B). Genotypic analysis of embryo tails separated from heads prior to Alcian blue staining confirmed that 6/6 embryos like that shown in Fig 4B were *wdr68*^*hi3812/hi3812*^ mutants. DM-treated wildtype (+/*) embryos appeared the same as DMSO-treated wildtypes (compare Fig 4C to 4A). However, DM-treated *wdr68*^*hi3812/hi3812*^ mutants lost M and also had severely reduced PQ cartilages while vehicle treated mutants displayed only joint fusions between M and PQ (compare Fig 4D to 4B). Genotypic analysis confirmed that 6/6 embryos like that shown in Fig 4D were *wdr68*^*hi3812/hi3812*^ mutants. We also quantified the fraction of mutants in each treatment exhibiting the mild M-PQ fusion and found that 23.2% of DMSO-treated animals displayed the M-PQ fusion versus only 4.2% of the DM-treated animals (Fig 4E, blue bars, p<0.046). Conversely, we found that only 3.7% of DMSO-treated animals displayed severe losses of M and PQ versus 20.0% of DM-treated animals (Fig 4E, orange bars, p<0.040).

To test whether partial inhibition of BMP signaling in mild *wdr68*^*hi3812/hi3812*^ embryos would yield a synergistic reduction of *edn1* expression, we again treated embryos raised at 24°C with DM starting at the 14–15 somites stage. We then used ISH to observe expression of *edn1* at the 20 somites stage. In embryos treated with DMSO, we found normal expression of *edn1* in wildtype (+/*) siblings (Fig 4F, red arrowhead) and reduced but still barely detectable expression of *edn1* in *wdr68*^*hi3812/hi3812*^ mutants (Fig 4G, red arrowhead). Genotypic analysis confirmed that 7/7 embryos in Fig 4F were wildtype (+/*) and 5/5 embryos shown in Fig 4G were *wdr68*^*hi3812/hi3812*^ mutants. In embryos treated with DM, we found mildly reduced expression of *edn1* in wildtype (+/*) siblings (Fig 4H, red arrowhead) that is consistent with the known role of DM as a BMP signaling antagonist (Yu et al., 2008) and with the fact that *edn1* is a downstream target of BMP signaling (Alexander et al., 2011; Zuniga et al., 2011). In the DM-treated *wdr68*^*hi3812/hi3812*^ mutants, we were unable to detect *edn1* expression (Fig 4I, red arrowhead). Genotypic analysis confirmed that 8/8 embryos shown in Fig 4H were wildtype (+/*) and that 5/5 embryos shown in Fig 4I were *wdr68*^*hi3812/hi3812*^ mutants.

### A BMP agonist can partially rescue Meckel’s cartilage formation and *edn1* expression in *wdr68* mutants

**Fig 5:**
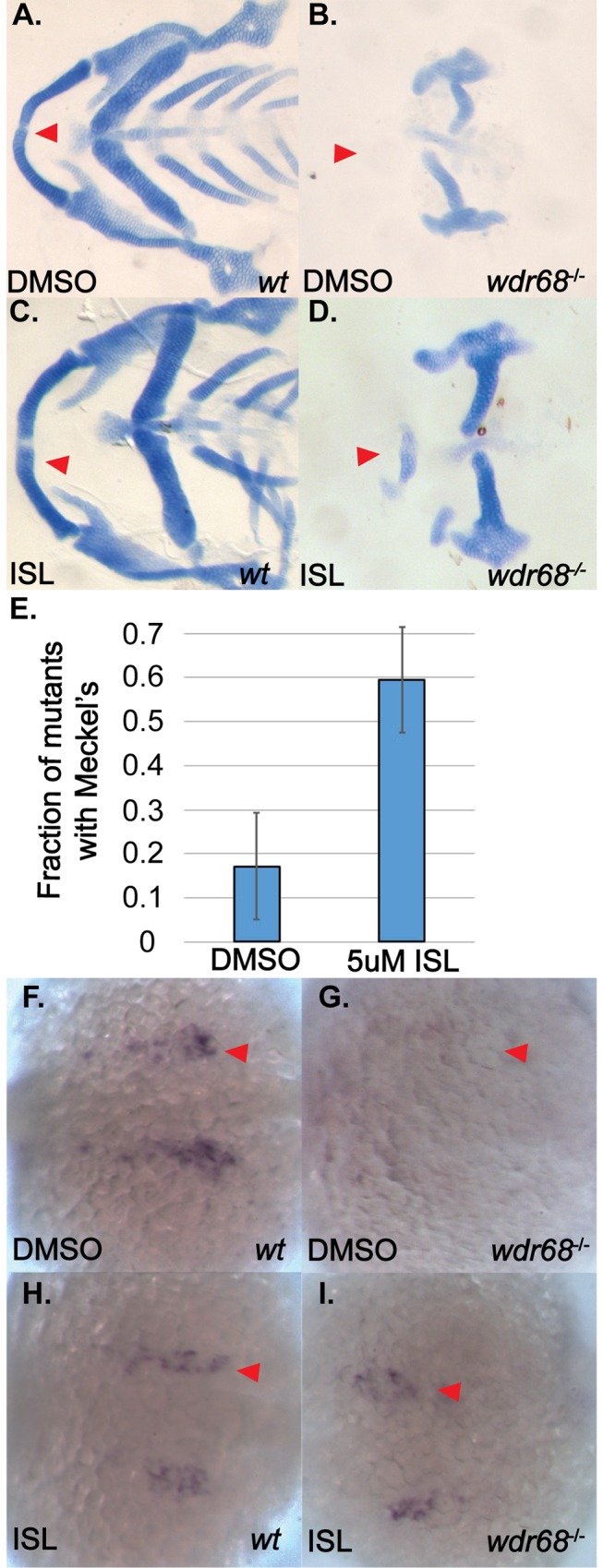
ISL treatment partially rescues M cartilage and *edn1* expression in *wdr68*^hi3812/hi3812^ zebrafish. (A-D) Flatmounts of 5dpf ventral cartilages of Alcian stained zebrafish raised at 32°C and treated with DMSO or 5μM ISL starting at the 14- to 15-somites stage. A) Wildtype zebrafish treated with DMSO control. Red arrow indicate M. B) *wdr68*^*hi3812/hi3812*^ mutants treated with DMSO control show a lack of M cartilage. C) Wildtype zebrafish treated with 5μM ISL show normal craniofacial cartilage formation. D) *wdr68*^*hi3812/hi3812*^ mutants treated with 5μM ISL show a partial rescue of M. E) Fraction of mutant embryos with partial M is significantly greater in the ISL treated group (p<0.006). (F-I) Dorsal views of *edn1* ISH analysis on 20-somites stage embryos treated with DMSO or 5μM ISL starting at the 14- to 15-somites stage. F) Wildtype embryos treated with DMSO control. G) *wdr68*^*hi3812/hi3812*^ mutants treated with DMSO control show lack of *edn1* expression. H) Wildtype embryos treated with ISL show similar expression compared to wild type. I) *wdr68*^*hi3812/hi3812*^ mutants treated with ISL are indistinguishable from that of wildtype.

If *wdr68* facilitates BMP induction of *edn1*, then treating *wdr68* mutants with a BMP agonist should at least partially rescue M cartilage formation and *edn1* expression. To test this, we treated embryos raised at 32°C with the BMP signaling agonist isoliquiritigenin (ISL) starting at the 14–15 somites stages [57]. Embryos were raised at 32°C so that mutants would exhibit severe loss of the M cartilage from which potential rescue of M cartilage formation could be most clearly assessed (see Fig 1C). Alcian blue stained zebrafish cartilages were dissected and flat mounted on slides for additional clarity. At 32°C, we found that DMSO-treated wildtype zebrafish developed the M cartilage as expected (Fig 5A, red arrowhead). Also at 32°C, we found that most DMSO-treated mutants exhibited complete loss of M and reduction of PQ as previously described (Fig 5B, red arrowhead). ISL-treated wildtype embryos were indistinguishable from DMSO-treated wildtype (compare Fig 5C to 5A). In contrast, ISL-treated mutants raised at 32°C exhibited a partial restoration of the M cartilage (compare Fig 5D to 5B, red arrowheads). However, ISL treatment did not appear to significantly restore formation of the PQ (compare Fig 5D to 5A and 5C). We quantified the fraction of mutants in each treatment exhibiting a discernible M-like cartilage element and found that 59% of ISL-treated animals displayed an M-like cartilage versus only 17% of DMSO-treated animals (Fig 5E, p<0.006).

To determine whether enhanced BMP signaling in severe *wdr68*^*hi3812/hi3812*^ embryos would partially rescue *edn1* expression, we again treated embryos raised at 32°C with 5μM ISL starting at the 14–15 somites stages. We then used ISH to observe expression of *edn1* at the 20 somites stage. In embryos treated with DMSO, we found normal expression of *edn1* in wildtype siblings (Fig 5F) but were unable to detect *edn1* expression in *wdr68*^*hi3812/hi3812*^ mutants (Fig 5G). Genotypic analysis confirmed that 10/10 embryos phenotypically like that shown in Fig 5F were wildtype (+/*) and 5/5 embryos phenotypically like that shown in Fig 5G were *wdr68*^*hi3812/hi3812*^ mutants. Strikingly in embryos treated with ISL, no embryos displayed a severe reduction of *edn1* expression. Genotyping was performed to unequivocally identify wildtypes and mutants. Wildtype siblings displayed normal expression of *edn1* (compare Fig 5H to 5F). The known BMP agonist role for ISL suggested we might see overexpression of *edn1* in these animals but that was not observed (Vrijens et al., 2013). Instead, the ISL-treated embryos appeared to yield no phenotypic mutants with respect to *edn1* expression level (compare Fig 5I to 5G). Consistent with that notion, random genotypic analysis revealed that 2/9 embryos with normal *edn1* expression levels were indeed *wdr68*^*hi3812/hi3812*^ mutants (Fig 5I). Thus, ISL treatment rescued *edn1* expression in *wdr68*^*hi3812/hi3812*^ mutants to near-normal levels.

Live confocal imaging of *Tg(sox10*:*mCherryCAAX);wdr68*^*hi3812/hi3812*^ mutants revealed consistent defects (6/6 mutants genotyped) within the 1^st^ arch CNCC region at the prim-5 and later stages (S5D, S5F and S5H Fig, blue dotted outline). At the prim-5 stage, reduced mCherryCAAX signal was observed in both dorsal and ventral regions of the 1^st^ arch (S5D Fig, compare blue outline to S5C Fig). By the prim-12 stage, the dorsal defect was less apparent but the ventral reduction was still evident (S5F Fig, compare blue outline to S5E Fig) and persisted through at least the prim-25 stage (S5H Fig, compare blue outline to S5G Fig). The mCherryCAAX signal remained detectable through to 4dpf (S5I–S5L Fig). ISL treatment yielded a modest but consistent (4/4 mutants genotyped) rescue of mCherryCAAX signal in the ventral region of the 1^st^ arch (S5N Fig, compare blue outline to S5H Fig).

### Canonical BMP signaling is not impaired in cells lacking Wdr68 expression

**Fig 6:**
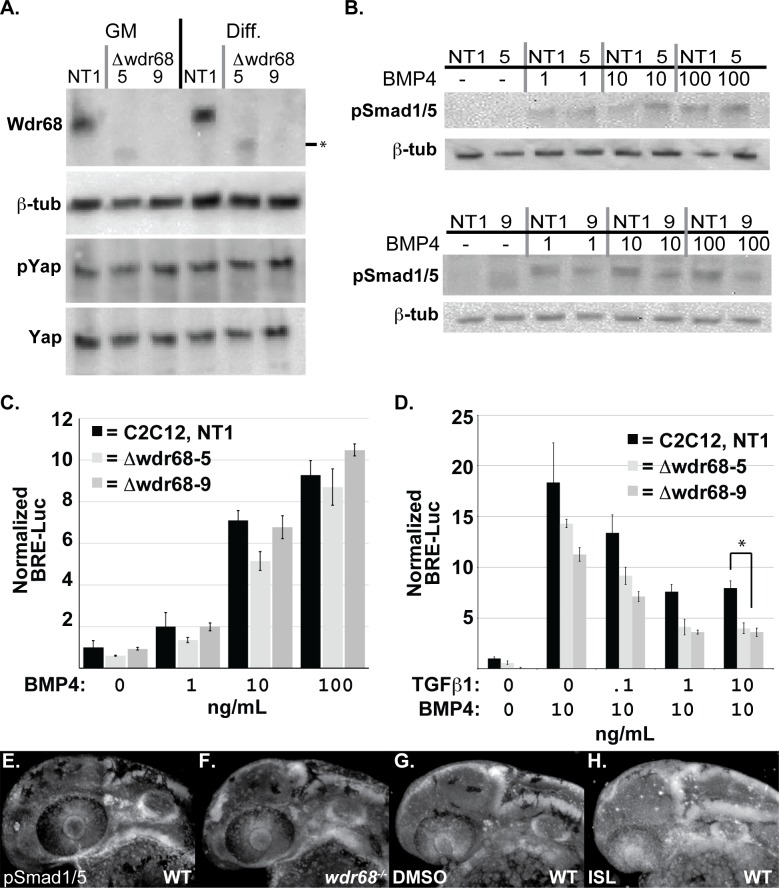
TGF-β interference with BMP signaling is enhanced in cells lacking Wdr68 expression. A) Isolation of Wdr68/Dcaf7 knock-out C2C12 cell sublines and expression levels in growth medium (GM) versus differentiation medium (Diff). Panel A1) Lanes 1 and 4, Wdr68 protein was detected in the control NT1 cells. Lanes 2 and 5, Δwdr68-5 lacks wildtype Wdr68 protein expression. Lanes 3 and 6, *Δwdr68*-9 lacks wildtype Wdr68 protein expression. Panel A2) β-tubulin expression was used as a loading control and did not differ substantially between lanes. Panel A3) pYap1 levels did not differ substantially between lanes. Panel A4) Total Yap1 levels did not differ substantially between lanes. B) pSmad1/5 induction was not substantially altered in *Δwdr68*-5 or *Δwdr68*-9 sublines. Panel B1) pSmad1/5 levels in control (NT1) or *Δwdr68*-5 (5) cells after 1 hour of exposure to 0, 1, 10, or 100ng/mL BMP4 in DM. Panel B2) β-tubulin expression was used as a loading control and did not differ substantially between lanes. Panel B3) pSmad1/5 levels in control (NT1) or *Δwdr68*-9 (5) cells after 1 hour of exposure to 0, 1, 10, or 100ng/mL BMP4 in DM. Panel B4) β-tubulin expression was used as a loading control and did not differ substantially between lanes. C) Transient transfection of NT1, *Δwdr68*-5, and *Δwdr68*-9 sublines with BRE-Luc and SV40-Renilla plasmids and induced with 0, 1, 10, or 100ng/mL BMP4 in GM. No significant differences were found between control and deletion sublines. Representative experiment shown from at least 3 independent trials. D) Transient transfection of NT1, *Δwdr68*-5, and *Δwdr68*-9 sublines with BRE-Luc and SV40-Renilla plasmids, induced with 10ng/mL BMP4, and then challenged with 0, 0.1, 1.0, or 10ng/mL TGF-®1. At 10ng/mL TGF-®1 interference with BRE-Luc activity was significantly greater in the *Δwdr68*-5 and *Δwdr68*-9 sublines relative to NT1 controls (* = p < 0.002). Representative experiment shown from at least 3 independent trials. E-H) Immunofluorescence detection of pSmad1/5 in prim-12 stage zebrafish embryos raised at 32°C. E) wildtype sibling embryo. F) *wdr68*^*hi3812/hi3812*^ mutant embryo. G) DMSO-treated wildtype sibling. H) ISL-treated wildtype sibling.

To further examine whether Wdr68 might directly impact BMP signaling, we used CRISPR/Cas9 gene targeting technology to generate loss-of-function deletions in the Wdr68/Dcaf7 locus in mouse C2C12 cells. Western blot analysis and DNA sequencing confirmed the generation of two independently isolated mutant sublines, *Δwdr68-5* and *Δwdr68-9* (Fig 6A and S4B Fig) as well as a non-target control (NT1) subline. The transcriptional co-activator Yap is reported to be important for BMP signaling in mammalian cells [58, 59]. In flies, the ortholog of Wdr68, Riquiqui *(*Riq*)*, is also reported to positively regulate the Yap ortholog *yorkie* through its interaction with the kinase Minibrain (Mnb) that negatively regulates the Hippo signaling pathway kinase Warts (Wts) [60]. Therefore, we examined both total Yap and pYap levels in the control, *Δwdr68-5*, and *Δwdr68-9* sublines but found no significant differences between them (Fig 6A). To further characterize the sublines, we examined the levels of pSmad1/5 after a one-hour treatment of the cells with 0, 1, 10, or 100ng/mL BMP4 and found no significant reproducible differences between the sublines (Fig 6B). To determine whether Wdr68/Dcaf7 might generally facilitate BMP signaling in a functional assay, we transfected the control and deletion sublines with the BRE-Luc reporter plasmid [55] along with a SV40-Renilla plasmid, and generated a dose-response curve to BMP4 ligand (Fig 6C). All relative light unit responses were normalized to the vehicle-treated control subline (Fig 6C, leftmost column). Overall, no significant decreases in fold-induction were found in the *Δwdr68-5* and *Δwdr68-9* sublines relative to the control subline (Fig 6C grey bars versus black bars). Immunofluorescence analysis also revealed little consistent difference in pSmad1/5 levels between wildtype and *wdr68*^*hi3812/hi3812*^ mutant zebrafish embryos (Fig 6E and 6F). As expected, ISL treatment did consistently increase pSmad1/5 signal (compare Fig 6H to 6G). Thus, Wdr68/Dcaf7 does not appear to directly modulate BMP signaling pathway activity.

### TGF-β interference with BMP signaling is enhanced in cells lacking Wdr68 expression

TGF-β signaling is important for craniofacial development [26], and can interfere with BMP signaling via Smad3-mediated displacement of Smad4 from pSmad1/5 complexes [29, 30]. Therefore we examined the BRE-Luc responsiveness of the NT1 control, *Δwdr68-5* and *Δwdr68-9* sublines when co-treated with 10ng/mL BMP4 and 0, 0.1, 1, or 10ng/mL TGF-β1 ligand. As previously reported, we found that TGF-β1 can interfere with BRE-Luc activity in the NT1 control cells (Fig 6D, black bars). We also found a consistent statistically significant further decline of BRE-Luc activity in the *Δwdr68-5* and *Δwdr68-9* sublines at 10ng/mL TGF-β1 (Fig 6D, grey bars versus black bars, p<0.001).

### The TGF-β inhibitor SB431542 can partially rescue the M cartilage defects and *edn1* expression in *wdr68* mutants

**Fig 7:**
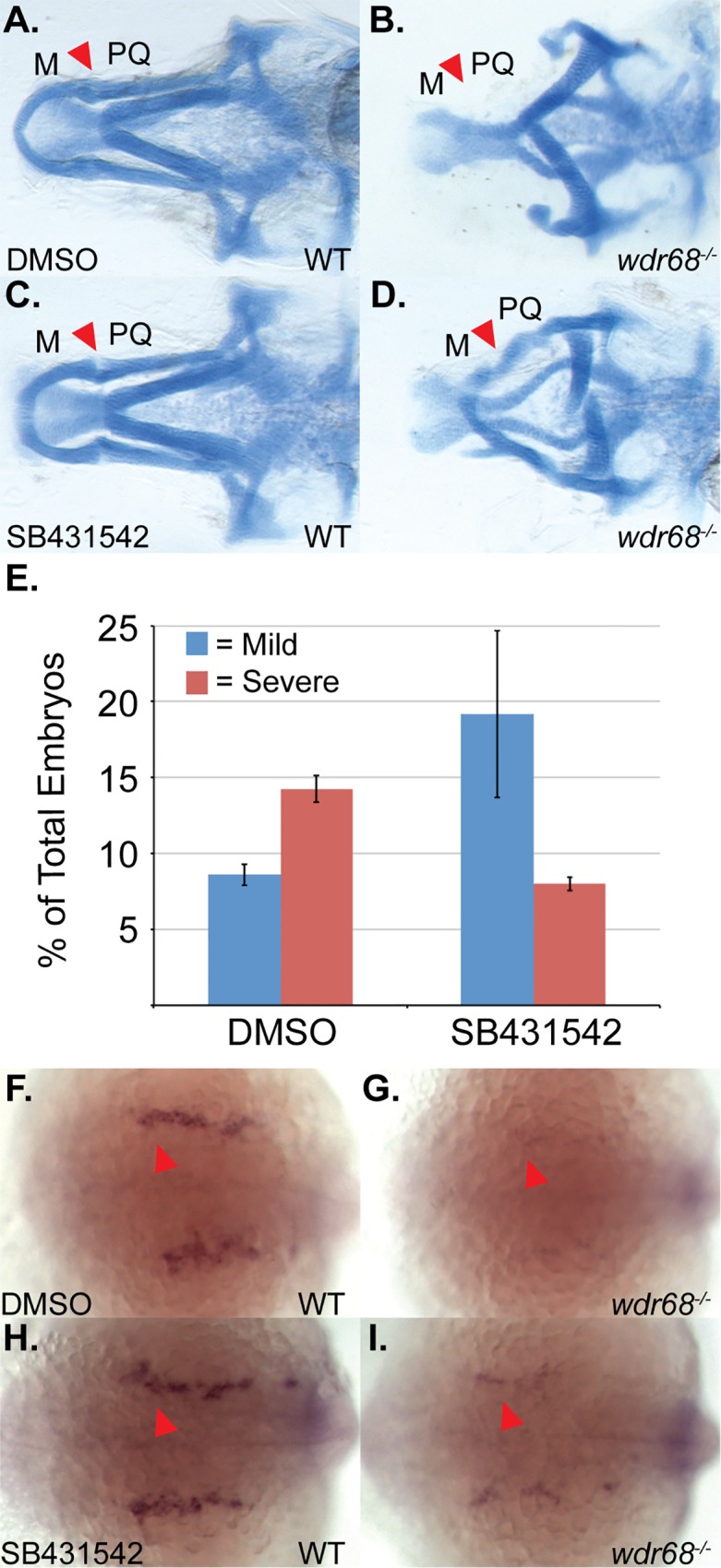
Inhibition of TGF-β signaling partially rescues M cartilage and *edn1* expression in *wdr68*^hi3812/hi3812^ zebrafish. (A-D) Ventral views of 5dpf Alcian stained craniofacial cartilages of zebrafish raised at 32°C and treated with DMSO or 10μM SB431542 at 14- to 15-somites stage. A) Wildtype zebrafish treated with DMSO control. Red arrow indicates M-PQ joint region. B) *wdr68*^*hi3812/hi3812*^ mutants treated with DMSO control show a lack of M cartilage. C) Wildtype zebrafish treated with 10μM SB431542 show normal craniofacial cartilage formation. D) *wdr68*^*hi3812/hi3812*^ mutants treated with 10μM SB431542 show a partial rescue of M. E) SB431542-treated mutants show a significantly reduced fraction of severe defects compared to the control group (p<0.012). (F-I) Dorsal views of *edn1* ISH analysis on 22-somites stage embryos treated with DMSO or 10μM SB431542 starting at the 14- to 15-somites stage. F) Wildtype embryos treated with DMSO control. G) *wdr68*^*hi3812/hi3812*^ mutants treated with DMSO control show lack of *edn1* expression. H) Wildtype embryos treated with SB431542 show similar expression compared to wild type. I) *wdr68*^*hi3812/hi3812*^ mutants treated with SB431542 show partial restoration of *edn1* expression.

If enhanced TGF-β interference with BMP signaling contributes to the *wdr68* mutant phenotype in zebrafish, then inhibiting TGF-β pathway function should at least partially restore cartilage development in *wdr68* mutants. SB431542 is a well-characterized inhibitor of the TGF-β family receptor kinases Alk4/5 [61], that is also known to inhibit TGF-β signaling activity in the zebrafish [62–64]. To avoid perturbing earlier developmental roles of TGF-β family signaling, embryos were treated with 10μM SB431542 starting at the 14- to 15-somites stages. Embryos were raised at 32°C so that mutants would exhibit severe loss of the M cartilage from which potential rescue of M cartilage formation could be most clearly assessed (Fig 1C). At 32°C, we found that DMSO-treated wildtype zebrafish developed the M cartilage as expected (Fig 7A, red arrowhead). Also at 32°C, we found that most DMSO-treated mutants exhibited complete loss of M and reduction of PQ as previously described (Fig 7B, red arrowhead). 10μM SB431542-treated wildtype embryos were indistinguishable from DMSO-treated wildtypes (compare Fig 7C to 7A). In contrast, SB431542-treated mutants raised at 32°C exhibited a partial restoration of the M and PQ cartilages (compare Fig 7D to 7B, red arrowheads). Quantitation of the changes in phenotypic severity revealed a significant shift in the number of mutants displaying severe versus mild phenotypes. Under DMSO treatment, mild *wdr68*^*hi3812/hi3812*^ embryos were 8.6% of the total sample population whereas severe *wdr68*^*hi3812/hi3812*^ embryos were 14.3% of the total population (Fig 7E, left). Under SB431542 treatment, mild *wdr68*^*hi3812/hi3812*^ embryos were 19.2% of the total population whereas severe mutants were 8.0% of the total population (Fig 7E, right). Thus, SB431542 treatment decreased the fraction of severe mutants relative to the DMSO control (Fig 7E, red bars, p < 0.012).

To determine whether inhibiting TGF-β signaling in severe *wdr68*^*hi3812/hi3812*^ embryos would partially rescue *edn1* expression, we again treated embryos raised at 32°C with 10μM SB431542 starting at the 14- to 15-somites stages. We then used ISH to observe expression of *edn1* at the 22-somites stage. In embryos treated with DMSO, we found normal expression of *edn1* in wildtype siblings (Fig 7F) but were unable to detect *edn1* expression in *wdr68*^*hi3812/hi3812*^ mutants (Fig 7G). Genotypic analysis confirmed that 4/4 embryos phenotypically like that shown in Fig 7F were wildtype (+/*) and 5/5 embryos phenotypically like that shown in Fig 7G were *wdr68*^*hi3812/hi3812*^ mutants. In embryos treated with SB431542, we also found normal expression of *edn1* in wildtype siblings (Fig 7H). Importantly, SB431542-treated mutants displayed greater *edn1* expression levels than could be observed in DMSO-treated mutants (Fig 7I, compare to Fig 7G), albeit at levels clearly still lower than wildtype siblings (Fig 7I, compare to Fig 7H). Genotyping confirmed that 5/5 embryos phenotypically like that shown in Fig 7H were wildtype (+/*) and 5/5 embryos phenotypically like that shown in Fig 7I were *wdr68*^*hi3812/hi3812*^ mutants. Thus, SB431542-treatment partially rescued *edn1* expression in *wdr68*^*hi3812/hi3812*^ mutants.

### The TGF-β inhibitor SB431542 can partially rescue dorsal *dlx2a* expression in *wdr68* mutants

**Fig 8:**
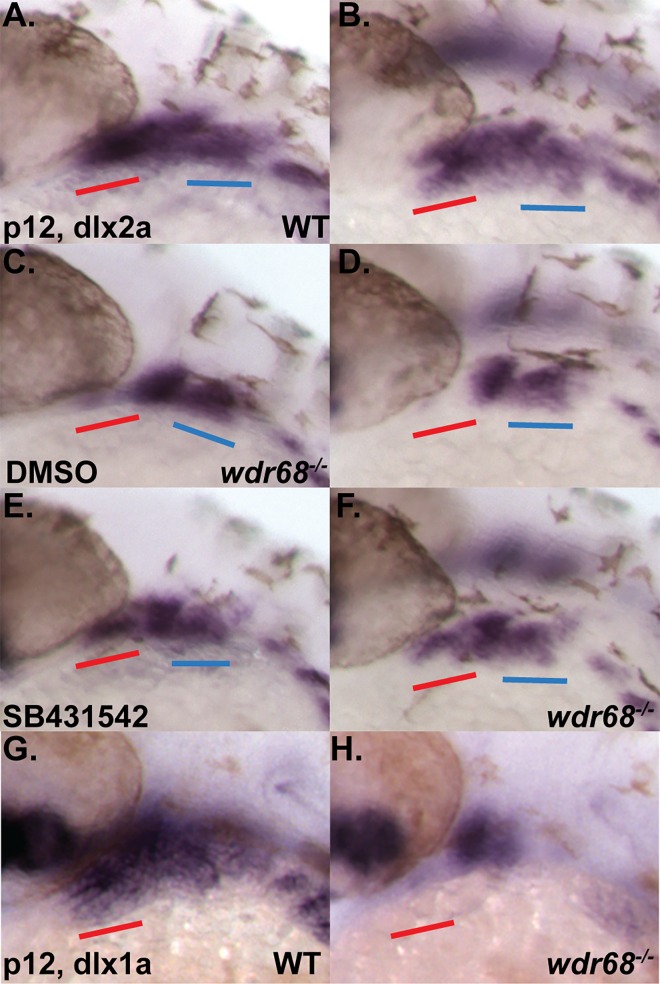
*dlx1a* and *dlx2a* expression is *wdr68-*dependent and responsive to inhibition of TGF-β signaling in *wdr68*^hi3812/hi3812^ zebrafish. (A-H) ISH analysis of prim-12 stage embryos raised at 32°C. A-F) *dlx2a* expression with red underline for anterior portion of 1^st^ arch and blue underline for 2^nd^ arch. A, C, E) lateral view. B, D, F) dorso-lateral view. A, B) DMSO-treated wildtype sibling showing normal *dlx2a*. C, D) DMSO-treated *wdr68*^*hi3812/hi3812*^ mutant showing loss of anterior 1^st^ arch *dlx2a*. E, F) SB431542-treated *wdr68*^*hi3812/hi3812*^ mutant showing partial rescue of anterior 1^st^ arch *dlx2a*. G, H) *dlx1a* expression. G) wildtype sibling showing normal *dlx1a*. H) *wdr68*^*hi3812/hi3812*^ mutant showing loss of anterior 1^st^ arch *dlx1a*.

A molecular explanation for the absence of the PQ in *wdr68*^*hi3812/hi3812*^ mutants is still lacking. Interestingly, SB431542-treated *wdr68*^*hi3812/hi3812*^ mutants not only displayed partial rescue of the ventral M cartilage, but also a consistent partial rescue of the dorsal PQ cartilage (Fig 7D). This was in contrast to either ectopic expression of *edn1* (Fig 2C), or ISL-treatment (Fig 5D), both of which failed to restore the dorsal PQ cartilage in *wdr68*^*hi3812/hi3812*^ mutants. It has been previously reported that simultaneous antisense knockdown of *dlx1a* and *dlx2a* causes loss of the PQ while retaining the M and PTP [65]. The reported *dlx1a/2a-*MO phenotype bears similarities to the residual defects we observed for both ectopic *edn1 (*Fig 2C) and ISL-treatment (Fig 5D) in *wdr68*^*hi3812/hi3812*^ mutants. Therefore, we re-examined the expression of the *dlx1a* and *dlx2a* genes at the prim-12 stage in *wdr68*^*hi3812/hi3812*^ mutant embryos raised at 32°C (Fig 8). In wildtype sibling embryos, we found robust expression of *dlx2a* in both dorsal and ventral CNCCs of the 1^st^ and 2^nd^ arches (Fig 8A and 8B). In contrast, we found severely reduced *dlx2a* expression in the anterior portion of the 1^st^ arch in *wdr68*^*hi3812/hi3812*^ mutants (Fig 8C and 8D, compare red underline between 8D and 8B). Consistent with the relatively minor 2^nd^ arch-derived cartilage defects in *wdr68*^*hi3812/hi3812*^ mutants (Figs 1C, 2B, 5B and 7B), expression of *dlx2a* in the 2^nd^ arch was relatively unchanged in *wdr68*^*hi3812/hi3812*^ mutants (Fig 8D compare blue underline to 8B). To determine whether inhibiting TGF-β signaling in severe *wdr68*^*hi3812/hi3812*^ embryos would partially rescue *dlx2a* expression, we again treated embryos raised at 32°C with 10μM SB431542 starting at the 14- to 15-somites stages. While SB431542 treatment yielded no discernible changes in wildtype siblings, we found partial rescue of anterior dorsal and ventral 1^st^ arch expression of *dlx2a* in *wdr68*^*hi3812/hi3812*^ mutants (Fig 8E and 8F, compare red underline to Fig 8C and 8D). Analysis of *dlx1a* expression revealed a similar loss of anterior dorsal and ventral 1^st^ arch expression in *wdr68*^*hi3812/hi3812*^ mutants (Fig 8H, compare red underline to Fig 8G).

## Discussion

### Temperature dependence of the *wdr68* mutant phenotype

Here we report that modest differences in maternally-derived protein stability, caused by incubation at different temperatures, can yield dramatic shifts in phenotypic severity even for mutants that are otherwise expected to be null mutations (Fig 1A–1D). This finding suggests that conducting forward genetic screens at temperatures other than the field standard of 28.5°C may reveal additional mutable loci that have as yet gone undetected.

### *wdr68* functions upstream of *edn1* during a period between the 17-somites and prim-5 stages to induce and restrict dorsal-specifying genes from ventral territory

We identified a developmental period between the 17-somites and prim-5 stages during which *wdr68* function is particularly important for craniofacial development (Fig 1D and 1E). Notably, this window contains the point at which *edn1* expression begins, around the 18-somites stage [18], and overlaps substantially with the period during which Edn1 signaling is important for craniofacial patterning [9, 10]. Specifically, Alexander et al., 2011 found that restoring Edn1 expression as late as the prim-5 stage was able to partially rescue the defects caused by dnBmpr1 expression. In contrast, restoring Edn1 expression around the prim-25 stage was too late, similar to our findings with Wdr68. We found that ectopic expression of *edn1* in *wdr68* mutants was able to partially restore the 1^st^ arch expression of a canonical *edn1-*dependent target gene, *dlx6a*, consistent with the model that *wdr68* functions upstream of *edn1* (Fig 2). However, we also found that ectopic *edn1* expression was insufficient to restore 1^st^ arch *hand2* expression in the absence of *wdr68 (*S3 Fig). The expression of *hand2* depends on both *edn1→dlx6a* and BMP signaling [9]. Thus, interference with BMP signaling to *hand2* in a *wdr68* mutant might prevent *edn1→dlx6a* induction of *hand2*. As an attempt to test this model, we treated *wdr68* mutants with ISL to enhance BMP signaling and *edn1* expression simultaneously, but the treatment failed to restore *hand2* expression (S4C and S4D Fig). While that result may simply reflect a shortcoming of the experimental procedure (e.g. insufficient levels of BMP signaling, *edn1→dlx6a* expression, or both), it may also indicate a distinct *wdr68-*dependent requirement for *hand2* expression. Nonetheless, and consistent with the known role for *edn1* expression, we found that *wdr68* is required to properly restrict *jag1b*, *hey1*, and *grem2* from ventral territory (Fig 3). The simplest explanation for these ventral expansions of dorsal territory markers is the failure of *edn1* expression in *wdr68* mutants.

The expansion of dorsal-specifying markers into ventral territory (Fig 3) is often accompanied by transformation of ventral cartilages into dorsal cartilages [9, 10]. Curiously, such transformations are not observed in *wdr68* mutants, which instead display losses of both dorsal and ventral cartilage elements. Our findings on the *wdr68-*dependence of *dlx1a* and *dlx2a* expression suggests that *jag1b-*mediated transformation of CNCCs may require the *dlx1a/2a* expression program.

The losses of *dlx1a/2a* expression (Fig 8) combined with the observed reductions of Tg(sox10:mCherryCAAX) label in *wdr68* mutants (S5 Fig) raises the possibility that a subset of the CNCCs in *wdr68* mutants may be lost due to apoptosis. This is an intriguing possibility that will require further investigation. However, the detection of dorsal-specifying markers such as *jag1b* and *hey1* in both dorsal and ventral territory (Fig 3) indicates that a population of CNCCs remains within the 1^st^ arch of *wdr68* mutants at least until about the prim-25 stage. The ultimate fate of the mis-patterned cells remains unclear.

### *wdr68* function does not directly modulate BMP signaling but may interfere with TGF-β signaling

Although a BMP antagonist and agonist yielded expected exacerbations and rescues of the defects in *wdr68* mutants, respectively (Figs 4 and 5), we found no strong evidence of a direct impact on BMP signaling pathway function (Fig 6). Notably, while analysis of the *edn1* promoter sequence revealed the presence of DNA sequence elements associated with BMP responsiveness (unpublished observations), it is unclear whether Smad1/5 complexes directly regulate the *edn1* promoter.

Interestingly, we found that TGF-β interference with BMP signaling was enhanced in mouse C2C12 cells lacking Wdr68 and that inhibition of TGF-β signaling partially restored jaw cartilage development, *edn1*, and *dlx2a* expression in zebrafish *wdr68* mutants (Figs 6D, 7 and 8). Nonetheless, is remains unclear whether these *wdr68—*TGF-β pathway interactions are direct or indirect.

The ISL-mediated rescue of *edn1* expression (Fig 5I) was more robust than the SB431542-mediated rescue of *edn1* expression (Fig 7I). Yet, ISL-mediated rescue of the jaw cartilages was less complete (Fig 5D) than the SB431542-mediated rescue of the jaw cartilages (Fig 7D). This discrepancy can be explained by the potency of *edn1* plus the absence of *dlx1a* and *dlx2a* expression in *wdr68* mutants. First, fairly low levels of *edn1* expression can still mediate M formation (Fig 4G). Second, SB431542 treatment was able to also rescue *dlx2a* (Fig 8E and 8F) while ISL treatment did not (S4E and S4F Fig). Thus, the combined actions of a relatively potent *edn1* signaling molecule and simultaneous restoration of *edn1* and *dlx2a* in SB431521-treated embryos likely underlies the relatively well formed jaw cartilages. That said, the mechanism by which SB431542 restores jaw development in *wdr68* mutants is still poorly understood and will require further investigation.

Wdr68 is a scaffolding protein and a large number of interacting factors have been identified by various high- and low-throughput approaches. Interestingly, Wdr68 has been reported to physically interact with Smad2, Smad3, and Ski [42, 66]. Wdr68 also interacts with the kinases Dyrk1a and Dyrk1b [38], that are reported to interact with Smad3 [42] and Ski [67], respectively. Thus, Wdr68 may organize a multi-subunit complex capable of modulating TGF-β pathway function. Together, the interactions of these factors potentially impact numerous disease states including cleft lip/palate, auriculocondylar syndrome, Alagille syndrome, and Down syndrome. Future investigations aimed at testing these potential roles are needed.

## Supporting Information

S1 Fig Detection of endogenous Wdr68 and ectopic GFP-Wdr68 fusion protein in zebrafish embryos. A-D) Immunofluorescence detection of endogenous Wdr68. A) Wdr68 expression in a wildtype 15-somites stage embryo raised at 32°C. B) a *wdr68* mutant lacking Wdr68 protein. C) Wdr68 expression in a wildtype prim-5 stage embryo raised at 32°C. D) a *wdr68* mutant lacking Wdr68 protein. E-F) live imaging of GFP fluorescence in *Tg(hsp70l*:*GFP-wdr68)*^*csu9*^ animals. E) transgenic animal with the only detected GFP expression coming from the *cmlc*:*eGFP* marker for transgenesis. F) same animal as in E but after a 0.5 hour heat shock (HS) at 39°C followed by 7 hours of recovery at 28.5°C. G) western blot analysis of *Tg(hsp70l*:*GFP-wdr68)*^*csu9*^ animals after various lengths of heat shock exposure. Panel G1) GFP-*wdr68* expression is induced by heat shock. Panel G2) β-tubulin expression was used as a loading control and did not differ substantially between lanes.
(TIF)

S2 Fig Phenotypes and distributions observed in ectopic GFP-Wdr68 fusion protein expressing embryos and control siblings. A-F) ventral views of 5dpf alcian blue stained cartilages from embryos raised at 28.5°C. A) heat shocked wildtype sibling displaying normal cartilages. B) heat shock induced *Tg(hsp70l*:*GFP-wdr68)* overexpression yielded no discernible cartilage phenotype. C) wdr68-MO injected animal showing the mild M-PQ joint fusion phenotype. D) wdr68-MO injected animal showing the severe loss of M and PQ phenotype. E) wdr68-MO injected heat shock induced *Tg(hsp70l*:*GFP-wdr68)* animal showing rescued normal M and PQ cartilages. F) wdr68-MO injected heat shock induced *Tg(hsp70l*:*GFP-wdr68)* animal showing rescued mild M-PQ joint fusions. G) plot of the distribution of phenotypes observed in a representative experiment on wdr68-MO injected *Tg(hsp70l*:*GFP-wdr68)* animals.
(TIF)

S3 Fig Epistatic analysis of *wdr68* and *edn1*. A-B) ISH analysis for *edn1* expression on 25-somites stage animals raised at 28.5C that were heat shocked at the bud and 20-somites stages. A) wildtype sibling with normal *edn1* expression. B) *Tg(hsp70l*:*GFP-wdr68)* embryo overexpressing GFP-Wdr68 with near-normal *edn1* expression. C-D) ISH analysis on prim-12 stage embryos. C) wildtype control sibling with normal *dlx6a* expression. D) Et1-MO;*wdr68*^*hi3812/hi3812*^ mutant lacking *dlx6a* expression in all arches. E-F) merged green-red channel fluorescence on prim-5 stage embryos injected with either GFP/dsRed (G/R) or GFP/Edn1 (G/Edn1) plasmid mixtures. E) broad GFP/dsRed expression in a G/R embryo. F) broad GFP expression in a G/Edn1 embryo. G-H) lateral view of 5dpf alcian blue stained embryo. G) Et1-MO injected animal showing loss of M and CH but retention of PQ. H) Et1-MO;*wdr68*^*hi3812/hi3812*^ mutant showing loss of M, CH, and PQ. I-P) ISH analysis for *hand2* expression on prim-12 stage animals raised at 32°C. Red arrowhead points at 1^st^ arch expression of *hand2*. I, K, M, O) dorsal view. J, L, N, P) lateral view. I, J) wildtype sibling injected with G/R mix showing normal *hand2*. K, L) *wdr68*^*hi3812/hi3812*^ mutant injected with G/R mix showing loss of 1^st^ arch *hand2*. M, N) wildtype sibling injected with G/Edn1 mix showing normal *hand2*. O,P) *wdr68*^*hi3812/hi3812*^ mutant injected with G/Edn1 mix showing loss of 1^st^ arch *hand2*.
(TIF)

S4 Fig BMP4, C2C12 deletions, and ISL controls. A) ISH analysis of prim-25 stage wildtype and wdr68 mutant animals revealed no differences in expression of *bmp4*. B) C2C12 wildtype and deletion subline sequences at the targeted locus in exon-5. The yellow highlight indicates the guide RNA target sequence followed by the TGG PAM sequence. C and D) ISH analysis for *hand2* expression. C) ISL-treated wildtype sibling. D) ISL-treated mutant sibling lacking rescue. E and F) ISH analysis for *dlx2* expression. E) ISL-treated wildtype sibling. F) ISL-treated mutant sibling lacking rescue.
(TIF)

S5 Fig Live confocal analysis of *Tg(sox10*:*mCherryCAAX);wdr68*^*hi3812/hi3812*^ mutants and wildtype siblings. A-N) Confocal images of the pharyngeal arch regions of embryos live-mounted in 0.7% agarose containing 0.0167% Tricaine. A) 25-somites stage wildtype sibling. B) 25-somites stage *wdr68*^*hi3812/hi3812*^ mutant. C) prim-5 stage wildtype sibling with 1^st^ arch region outlined in blue. D) prim-5 stage *wdr68*^*hi3812/hi3812*^ mutant with same outline as in C to indicate regions of reduced mCherryCAAX signal. E) prim-12 stage wildtype sibling with ventral 1^st^ arch region outlined in blue. F) prim-12 stage *wdr68*^*hi3812/hi3812*^ mutant with same outline as in E to indicate reduced ventral mCherryCAAX signal. G) prim-25 stage wildtype sibling with ventral 1^st^ arch region outlined in blue. H) prim-25 stage *wdr68*^*hi3812/hi3812*^ mutant with same outline as in G to indicate reduced ventral mCherryCAAX signal. I) lateral view of 4-dpf wildtype sibling cartilages. J) lateral view of 4-dpf *wdr68*^*hi3812/hi3812*^ mutant severely reduced M and PQ cartilages. K) ventral view of animal in I. L) ventral view of animal in J. M) ISL-treated prim-25 stage wildtype sibling with ventral 1^st^ arch region outlined in blue. N) ISL-treated prim-25 stage *wdr68*^*hi3812/hi3812*^ mutant with same outline as in M, H, G to indicate modest rescue of ventral 1^st^ arch mCherryCAAX signal.
(TIF)
