# Supplementary figures and images for: Wdr68 Mediates Dorsal and Ventral Patterning Events for Craniofacial Development
Source: PLoS One. 2016 Nov 23;11(11):e0166984. doi: 10.1371/journal.pone.0166984 (PMC5120840; doi:10.1371/journal.pone.0166984)

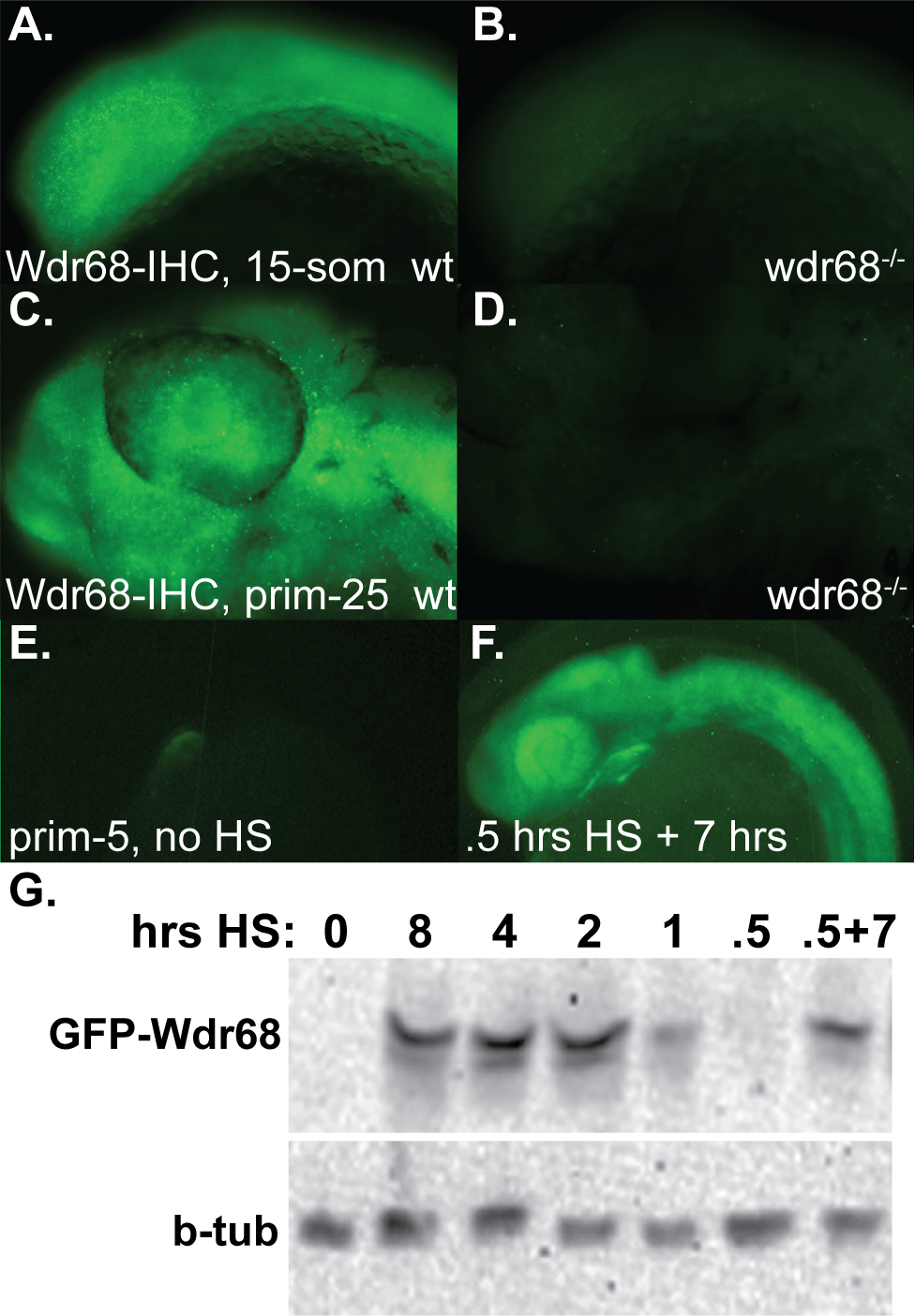

Supplement: S1 Fig — A-D) Immunofluorescence detection of endogenous Wdr68. A) Wdr68 expression in a wildtype 15-somites stage embryo raised at 32°C. B) a wdr68 mutant lacking Wdr68 protein. C) Wdr68 expression in a wildtype prim-5 stage embryo raised at 32°C. D) a wdr68 mutant lacking Wdr68 protein. E-F) live imaging of GFP fluorescence in Tg(hsp70l:GFP-wdr68)csu9 animals. E) transgenic animal with the only detected GFP expression coming from the cmlc:eGFP marker for transgenesis. F) same animal as in E but after a 0.5 hour heat shock (HS) at 39°C followed by 7 hours of recovery at 28.5°C. G) western blot analysis of Tg(hsp70l:GFP-wdr68)csu9 animals after various lengths of heat shock exposure. Panel G1) GFP-wdr68 expression is induced by heat shock. Panel G2) β-tubulin expression was used as a loading control and did not differ substantially between lanes. (TIF) [file pone.0166984.s001.tif]

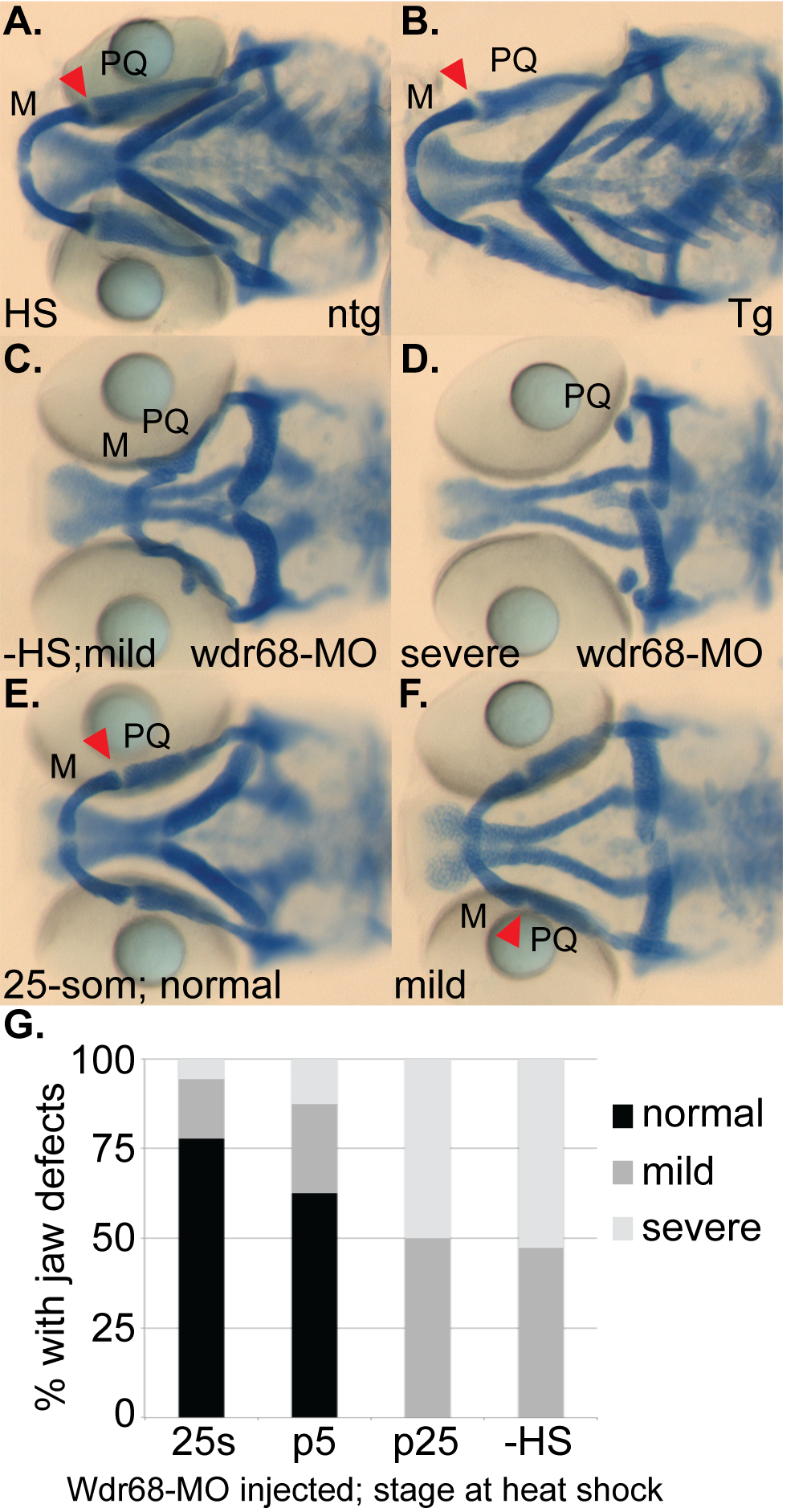

Supplement: S2 Fig — A-F) ventral views of 5dpf alcian blue stained cartilages from embryos raised at 28.5°C. A) heat shocked wildtype sibling displaying normal cartilages. B) heat shock induced Tg(hsp70l:GFP-wdr68) overexpression yielded no discernible cartilage phenotype. C) wdr68-MO injected animal showing the mild M-PQ joint fusion phenotype. D) wdr68-MO injected animal showing the severe loss of M and PQ phenotype. E) wdr68-MO injected heat shock induced Tg(hsp70l:GFP-wdr68) animal showing rescued normal M and PQ cartilages. F) wdr68-MO injected heat shock induced Tg(hsp70l:GFP-wdr68) animal showing rescued mild M-PQ joint fusions. G) plot of the distribution of phenotypes observed in a representative experiment on wdr68-MO injected Tg(hsp70l:GFP-wdr68) animals. (TIF) [file pone.0166984.s002.tif]

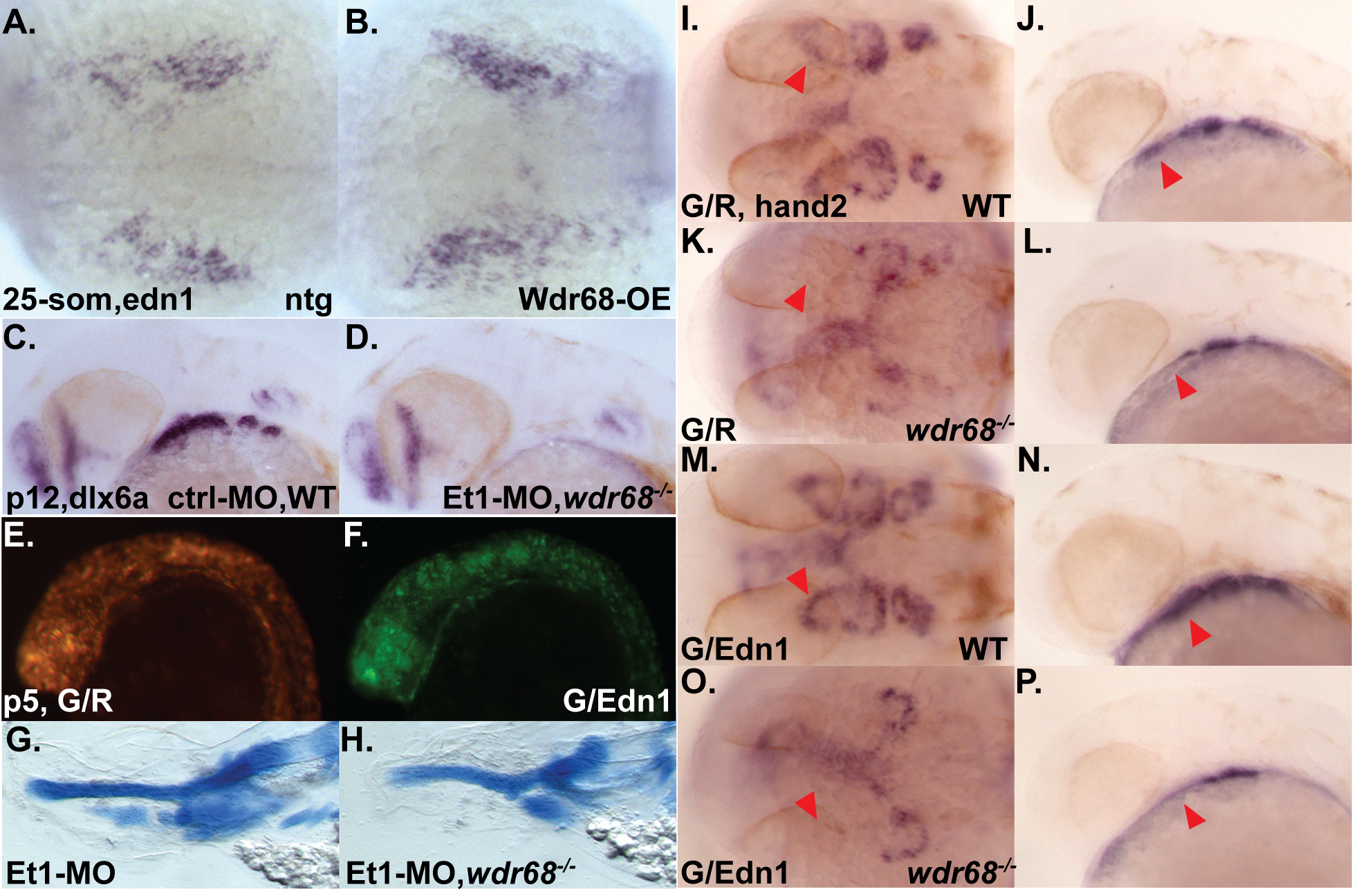

Supplement: S3 Fig — A-B) ISH analysis for edn1 expression on 25-somites stage animals raised at 28.5C that were heat shocked at the bud and 20-somites stages. A) wildtype sibling with normal edn1 expression. B) Tg(hsp70l:GFP-wdr68) embryo overexpressing GFP-Wdr68 with near-normal edn1 expression. C-D) ISH analysis on prim-12 stage embryos. C) wildtype control sibling with normal dlx6a expression. D) Et1-MO;wdr68hi3812/hi3812 mutant lacking dlx6a expression in all arches. E-F) merged green-red channel fluorescence on prim-5 stage embryos injected with either GFP/dsRed (G/R) or GFP/Edn1 (G/Edn1) plasmid mixtures. E) broad GFP/dsRed expression in a G/R embryo. F) broad GFP expression in a G/Edn1 embryo. G-H) lateral view of 5dpf alcian blue stained embryo. G) Et1-MO injected animal showing loss of M and CH but retention of PQ. H) Et1-MO;wdr68hi3812/hi3812 mutant showing loss of M, CH, and PQ. I-P) ISH analysis for hand2 expression on prim-12 stage animals raised at 32°C. Red arrowhead points at 1st arch expression of hand2. I, K, M, O) dorsal view. J, L, N, P) lateral view. I, J) wildtype sibling injected with G/R mix showing normal hand2. K, L) wdr68hi3812/hi3812 mutant injected with G/R mix showing loss of 1st arch hand2. M, N) wildtype sibling injected with G/Edn1 mix showing normal hand2. O,P) wdr68hi3812/hi3812 mutant injected with G/Edn1 mix showing loss of 1st arch hand2. (TIF) [file pone.0166984.s003.tif]

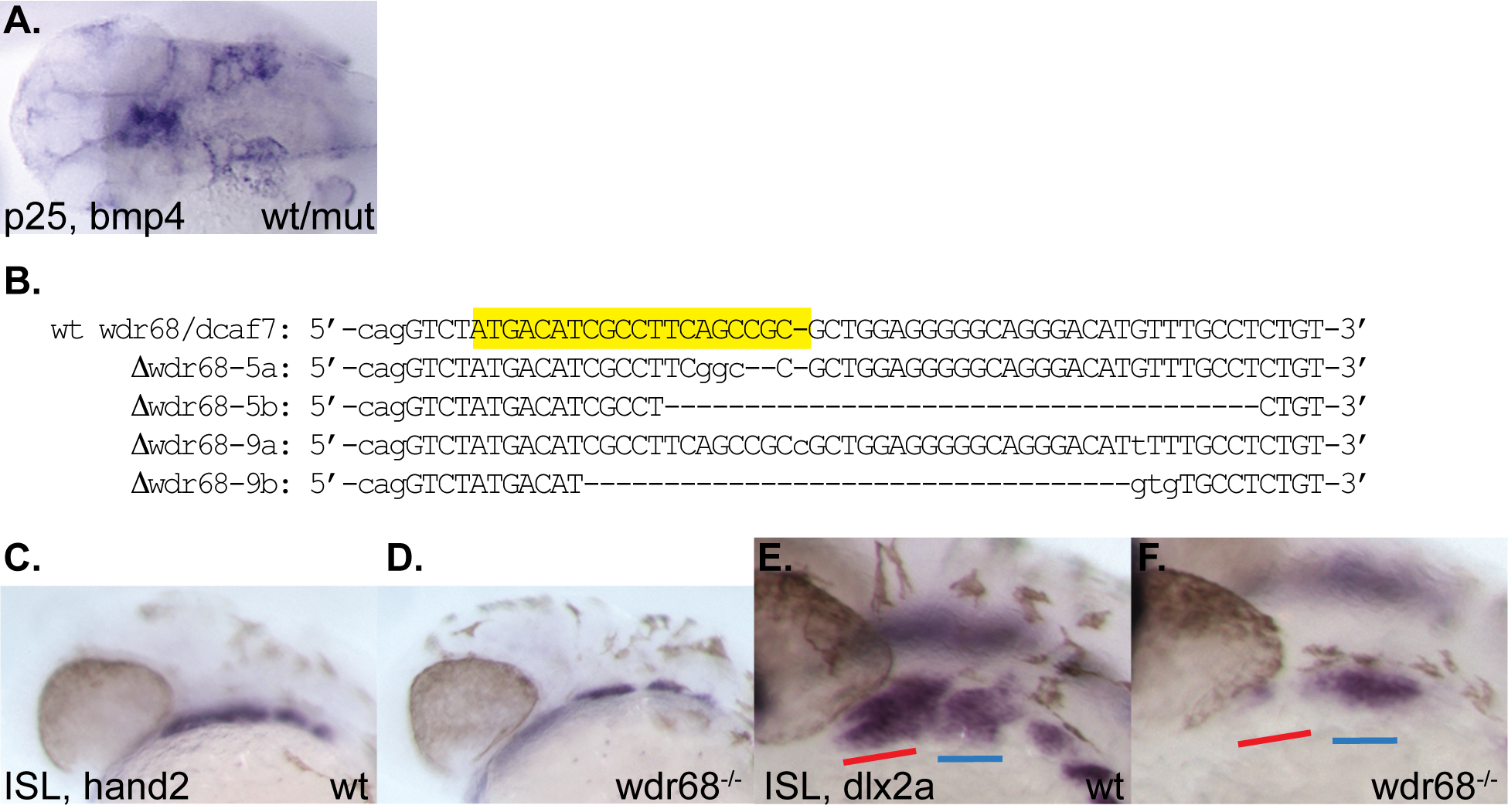

Supplement: S4 Fig — A) ISH analysis of prim-25 stage wildtype and wdr68 mutant animals revealed no differences in expression of bmp4. B) C2C12 wildtype and deletion subline sequences at the targeted locus in exon-5. The yellow highlight indicates the guide RNA target sequence followed by the TGG PAM sequence. C and D) ISH analysis for hand2 expression. C) ISL-treated wildtype sibling. D) ISL-treated mutant sibling lacking rescue. E and F) ISH analysis for dlx2 expression. E) ISL-treated wildtype sibling. F) ISL-treated mutant sibling lacking rescue. (TIF) [file pone.0166984.s004.tif]

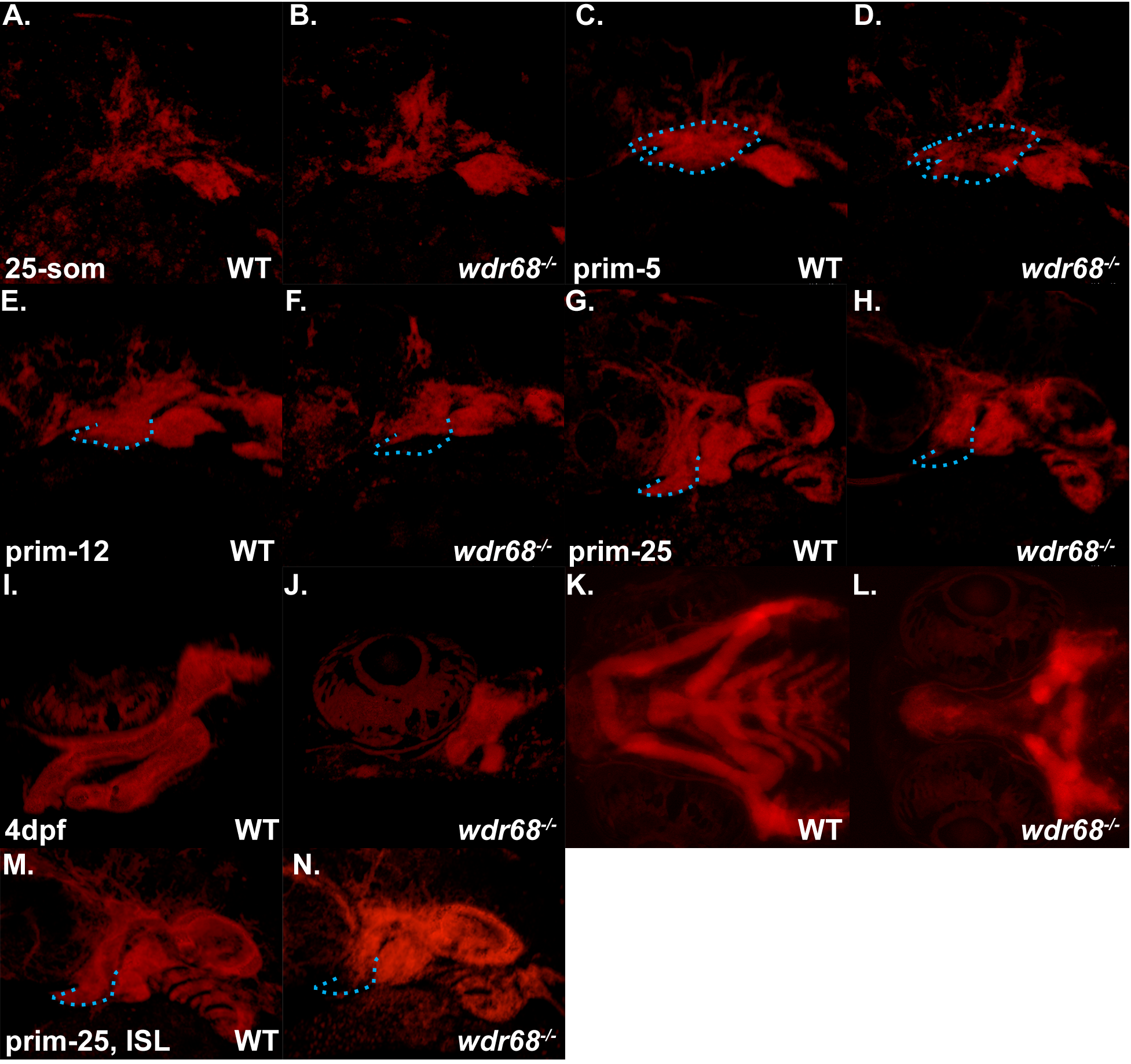

Supplement: S5 Fig — A-N) Confocal images of the pharyngeal arch regions of embryos live-mounted in 0.7% agarose containing 0.0167% Tricaine. A) 25-somites stage wildtype sibling. B) 25-somites stage wdr68hi3812/hi3812 mutant. C) prim-5 stage wildtype sibling with 1st arch region outlined in blue. D) prim-5 stage wdr68hi3812/hi3812 mutant with same outline as in C to indicate regions of reduced mCherryCAAX signal. E) prim-12 stage wildtype sibling with ventral 1st arch region outlined in blue. F) prim-12 stage wdr68hi3812/hi3812 mutant with same outline as in E to indicate reduced ventral mCherryCAAX signal. G) prim-25 stage wildtype sibling with ventral 1st arch region outlined in blue. H) prim-25 stage wdr68hi3812/hi3812 mutant with same outline as in G to indicate reduced ventral mCherryCAAX signal. I) lateral view of 4-dpf wildtype sibling cartilages. J) lateral view of 4-dpf wdr68hi3812/hi3812 mutant severely reduced M and PQ cartilages. K) ventral view of animal in I. L) ventral view of animal in J. M) ISL-treated prim-25 stage wildtype sibling with ventral 1st arch region outlined in blue. N) ISL-treated prim-25 stage wdr68hi3812/hi3812 mutant with same outline as in M, H, G to indicate modest rescue of ventral 1st arch mCherryCAAX signal. (TIF) [file pone.0166984.s005.tif]
